# Synergistic Ultramicropore and Hierarchical Pore Engineering in Heteroatom-Doped Carbon for High-Performance Zinc-Ion Capacitors

**DOI:** 10.1007/s40820-026-02181-0

**Published:** 2026-04-17

**Authors:** Jiale Zhang, Ruifang Zhang, Yangbo Du, Shuaihua Zhang, Runze Gao, Xuanqi Huang, Qi Yang, Debin Kong, Zhichang Xiao

**Affiliations:** 1 Department of Chemistry, College of Science, Hebei Agricultural University, Baoding, 071001 People’s Republic of China; 2 College of Land and Resources, Hebei Agricultural University, Baoding, 071001 People’s Republic of China; 3 Key Laboratory of Civil Aviation Thermal Disaster Control and Emergency, Civil Aviation University of China, Tianjin, 300300 People’s Republic of China; 4 Wuzhen Laboratory, Jiaxing, 314500 People’s Republic of China; 5 College of New Energy, China University of Petroleum (East China), Qingdao, 266580 People’s Republic of China

**Keywords:** Ultramicropore, Hierarchical pore, Carbonaceous zinc-ion capacitor, Desolvation effect, Air self-charging

## Abstract

Carbonaceous zinc-ion capacitors (ZICs) offer inherent advantages for energy storage, yet the role of pore structures in enabling high zinc-ion capacitance remains underexplored. Herein, a dual-molten-salt regulation strategy is employed to derive N/O/S-doped porous carbon nanomaterials, achieving a high specific surface area (SSA) of 2523 m^2^ g^−1^ with ultramicropores (< 0.86 nm) contributing 30.6% of the total SSA. Structural analyses reveal that increasing molten FeCl_3_ content yields materials with comparable heteroatom contents and defect structures, but a progressive shift from ultramicropores to mesopores. Crucially, the individual contributions of the pore structure are decoupled by both in situ characterizations and theoretical simulations: The ultramicropores facilitate the desolvation of [Zn(H_2_O)_6_]^2+^ (ultramicropore effect), while the hierarchical pores ensure rapid ion transport (hierarchical pore effect). The optimized HHPC-2 delivers a high specific capacitance of 222.6 F g^−1^ at 1 A g^−1^ and an energy density of 120.0 Wh kg^−1^ in ZICs. Intriguingly, its outstanding oxygen reduction reaction catalytic activity enables self-charging upon air exposure after a full discharge, achieving a self-charging rate of 15 mAh g^−1^ h^−1^ and recovering 80% of the externally charged capacity in subsequent discharge cycles. This positions the device as highly promising for practical deployment in regions with intermittent grid power supplies.

**Supplementary Information:**

The online version contains supplementary material available at 10.1007/s40820-026-02181-0.

## Highlights

- A series of N/O/S-doped porous carbons are synthesized through a dual-molten-salt regulation strategy with controllable ultramicropore and mesopore structures.
- The distinct functions of ultramicropores and hierarchical pores are delineated experimentally and theoretically, demonstrating that ultramicropores are crucial for facilitating the desolvation of [Zn(H_2_O)_6_]^2+^, while the hierarchical network ensures rapid ion transport.
- The optimized cathode delivers a high specific capacitance (336.9 F g^−1^), outstanding energy density (120.0 Wh kg^−1^) and excellent air self-charging capability.

**Supplementary Information:**

The online version contains supplementary material available at 10.1007/s40820-026-02181-0.

## Graphical abstract

**Figure:**
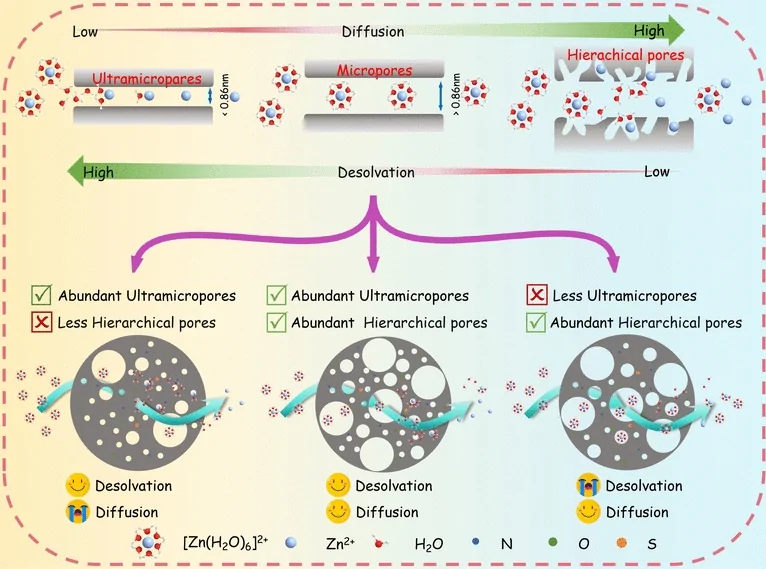

## Introduction

Under the strategic imperative of global energy transition, the demands for renewable energy storage and efficient energy conversion have become increasingly pronounced. Developing novel electrochemical energy storage technologies characterized by high safety, high energy density, long cycle stability, and environmental benignity has emerged as the pivotal pathway to surmount the bottlenecks in energy storage [1]. Lithium-ion batteries, leveraging their superior energy density, dominate applications in portable electronics and electric vehicles; however, they are hampered by technical limitations such as lithium resource scarcity, elevated costs, and inherent safety risks, thereby constraining their scalability for large-scale energy storage [2, 3]. In contrast, zinc metal serves as an exemplary anode material, endowed with salient advantages including a high theoretical specific capacity (820 mAh g^−1^), favorable redox potential (− 0.76 V vs. SHE), abundant reserves, and exceptional stability [4, 5]. In particular, carbon-based zinc-ion capacitors (ZICs) synergistically integrate the rapid anion adsorption/desorption kinetics of supercapacitors, together with the Faradic ion intercalation/deintercalation mechanisms of zinc-ion batteries [6, 7], positioning them as a promising avenue for next-generation large-scale energy storage systems. Nonetheless, the energy density of contemporary carbon-based ZICs remains inferior, chiefly attributable to the constrained zinc-ion storage capacitance of carbon cathodes relative to the elevated theoretical specific capacity of the anode—a limitation primarily stemming from their limited specific surface area (SSA) and surface functional groups.

Porous carbon nanomaterials principally facilitate charge storage via electrostatic adsorption/desorption of zinc ions within their pore architectures. Through precise pore structure engineering, the design of porous carbon nanomaterials with optimized pore size distributions can effectively curtail ion/electron transport and diffusion pathways while furnishing augmented SSA, thereby enhancing the rate capability and cycling stability of capacitors [8]. Conventionally, ultramicropores narrower than 0.86 nm are deemed ineffective for accommodating hydrated zinc ions (approximately 0.86 nm in size). For instance, Wang et al. [9] demonstrated the importance of matchable pore size by eliminating the presence of micropores through a delicate structural design, which eventually enhanced the zinc-ion storage capability of the optimized electrode materials. Furthermore, researchers have pivoted toward hierarchical pore architectures. Jian et al. [10] synthesized porous carbon nanosheets with pore sizes of 0.55, 0.65, and 0.75 nm, and molecular dynamics simulations corroborated that porous carbons enriched with a high proportion of mesopores and micropores exceeding 0.75 nm markedly elevate the specific capacitance and rate performance of ZICs. Zheng et al. [11] employed a coupled pre-carbonization and chemical activation strategy, harnessing the solubility of alkaline lignin and the in situ spatial confinement effect of K_2_CO_3_ to fabricate carbon materials featuring a micro-meso-macro hierarchical pore structure. This hierarchical design substantially improved the transport efficiency of hydrated zinc ions, facilitating rapid zinc-ion diffusion and manifesting superior rate capability and stability in ZICs. However, within the supercapacitor domain, the correlation between pore architecture and ZICs performance remains contentious. Previous investigations into ionic liquid capacitors revealed that pores smaller than the diameter of charge carriers can still store charge via electrostatic interactions [12], while the work of Hao et al. established structure–performance relationships that unequivocally elucidated the micropore effect in ionic liquid capacitors [13]. For ZICs, researchers have likewise begun probing the interplay between ultramicropores and zinc-ion storage performance. Recently, Kang et al. demonstrated that subnanopores can induce desolvation effects and enhance storage capacity [14]. From a chemical perspective, devising model systems with controllable pore architectures to delineate the influence of pore size on zinc-ion capacitive performance would furnish a critical platform for rationally advancing ZICs efficacy.

Meanwhile, heteroatom doping constitutes another pivotal strategy for augmenting the zinc-ion storage performance of porous carbon materials. The incorporation of heteroatoms (e.g., N, O, S, P) enables modulation of surface chemistry, enhancing the wettability and conductivity between carbon materials and electrolytes while refining surface charge distribution. Moreover, heteroatoms could function as Zn^2+^ storage sites, accelerating zinc-ion transmission kinetics and capacitance via redox reactions such as chemical adsorption [15–17]. Zhang et al. [18] adopted an ammonia treatment protocol to construct hierarchically porous carbon with 2.79 at% nitrogen content. The nitrogen incorporation enhanced cathode conductivity, hydrophilicity, and pseudocapacitive reaction kinetics, while promoting chemical adsorption of Zn^2+^ on the carbon surface, elevating the specific capacity from 67.8 to 177.8 mAh g^−1^. Carbonyl groups have been substantiated to enable high-capacity storage through reversible C–O–Zn bond formation [10], whereas oxygen heteroatoms enhance surface wettability to facilitate intimate contact with Zn^2+^. For sulfur heteroatom, its coordination with Zn^2+^ was demonstrated to be capable of improving conductivity and electrode hydrophilicity [19, 20], with ex situ X-ray photoelectron spectroscopy analyses indicating the formation of Zn–S species [21, 22], and computational simulations affirming that sulfur accelerates zinc-ion adsorption kinetics [23]. Moreover, multiheteroatom co-doping engenders synergistic effects that further elevate zinc-ion storage. For example, Zhang et al. [24] developed a supermolecule-mediated direct pyrolysis carbonization approach to yield three-dimensional, high-content heteroatom-doped porous carbon (N: 14.9 at%, O: 4.7 at%). This material exhibited high specific capacitance and rate performance in ZICs, with density functional theory calculations and molecular dynamics simulations revealing that edge nitrogen and oxygen dopants facilitate reversible adsorption/desorption of zinc ions and protons. Investigations confirm that rational heteroatom doping, in concert with suitable pore architectures, promotes desolvation of [Zn(H_2_O)_6_]^2+^, thereby enhancing zinc-ion storage kinetics. For instance, Ying et al. [25] engineered polyacrylonitrile-derived ultramicroporous carbon nanospheres with a uniform pore size of 0.59 nm, leveraging pore confinement to induce pre-desolvation of [Zn(H_2_O)_6_]^2+^; the strong coordinative adsorption of Zn^2+^ by C–P and C=O bonds, coupled with the hydrophobic nature of C–P bonds, lowered the desolvation energy barrier of [Zn(H_2_O)_6_]^2+^, expediting cation desolvation. Thus, the rational design of porous carbon materials integrating high heteroatom content and ultramicroporous structures to elevate zinc-ion storage performance warrants in-depth exploration.

Beyond the aforementioned challenges in enhancing storage capacitance and rate performance for carbon-based ZICs, their practical implementation is further impeded by limitations in scenario adaptability. On the one hand, in fields such as health monitoring and sports recording, the escalating requirements for battery portability, multifunctionality, and intelligence are driving the advancement of flexible integration for ZICs [26]. This paradigm shift requires electrode materials endowed with exceptional stability—both mechanical and chemical microstructural—to mitigate performance degradation. Mechanically, robust structural integrity is essential to preserve electrochemical performance during repeated bending cycles; and chemically, enduring microstructural fidelity ensures sustained high performance even after extensive long-term cycling. On the other hand, grid supply deficits in numerous regions impede charging processes of those rechargeable batteries. To circumvent this obstacle, researchers have explored oxidative strategies for chemical self-charging of fully discharged ZICs [27–29]. Notably, leveraging oxygen from the ubiquitous air to transduce its chemical energy into electrical energy within ZICs has emerged as a focal point, bearing profound implications for environmental and energy sustainability [30, 31]. To achieve high-performance self-charging of ZICs, two important targets are highly required: (i) elevated self-charging efficiency (self-charging capacity/galvanostatic charge–discharge capacity) and (ii) rapid self-charging rate (self-charging capacity/self-charging time) [32]. Principally, accelerating the oxygen reduction rate to re-oxidize discharged electrode materials constitutes the crux, thereby mandating electrode materials with exceptional oxygen reduction reaction (ORR) catalytic performance.

**Fig. 1:**
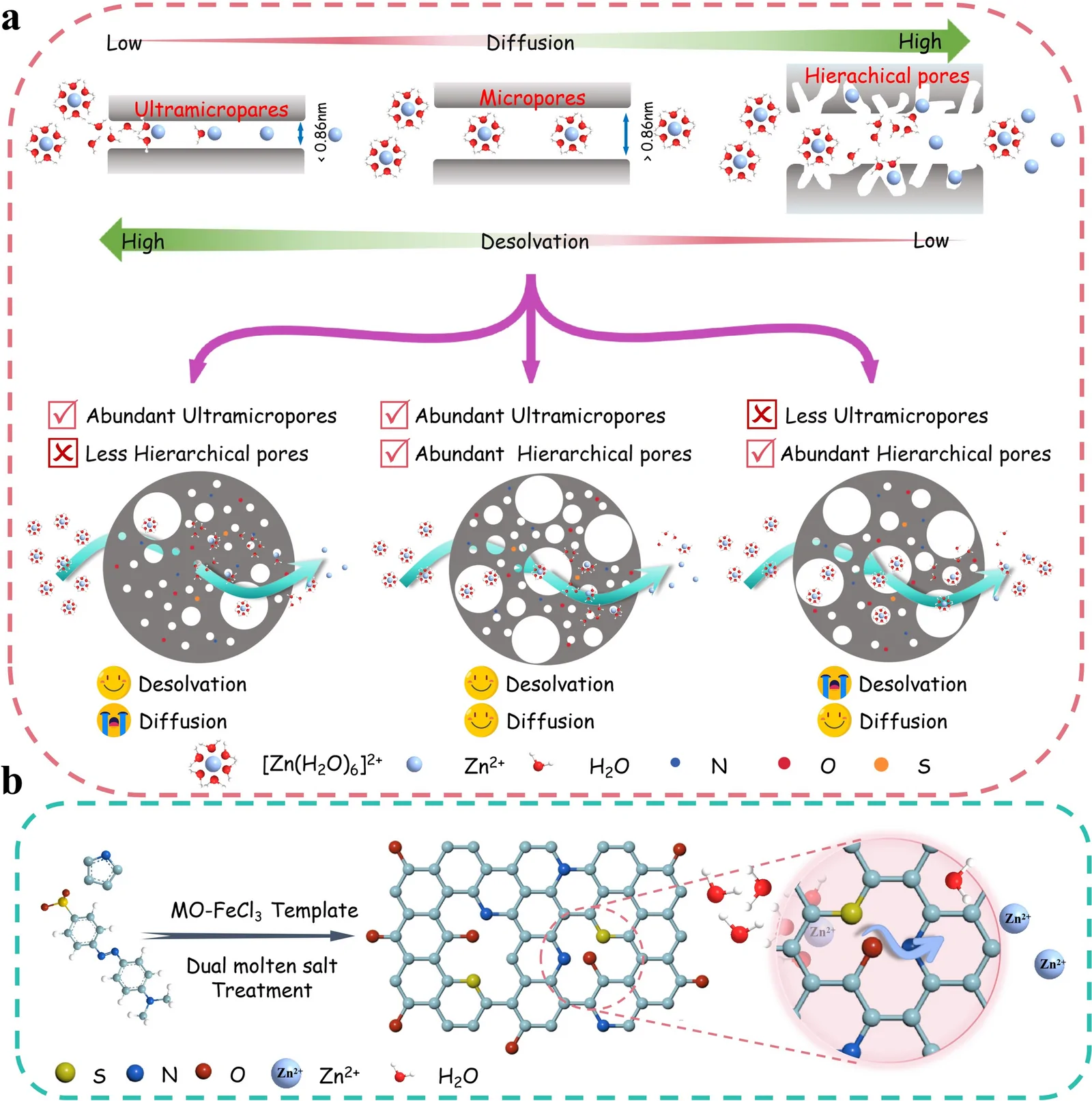
**a** Design principle for the porous structure with superior Zn^2+^ storage capacity and kinetics. **b** Schematic illustration for the preparation of HHPC-x and the desolvation process of [Zn(H_2_O)_6_]^2+^

To address the aforementioned dilemma, herein we develop a molten-salt-assisted pyrolysis strategy using polypyrrole as the precursor to construct heteroatom-doped hierarchical porous carbon materials (HHPC) that integrate N/S/O co-doping with ultramicroporous architectures. By precisely tuning the dual-molten-salt ratio, this approach enables tailored optimization of pore size distributions (both high ratio of ultramicropores and hierarchical pores) and surface chemistry modulation, thereby synergistically enhancing zinc-ion storage capacitance and transport dynamics (Fig. 1a). Density functional theory (DFT) calculations reveal that while the desolvation of [Zn(H_2_O)_6_]^2+^ is energetically unfavorable in larger pores, an ultramicropore of 0.7 nm induces steric confinement which alone can lower the desolvation barrier. Crucially, the incorporation of N/S/O heteroatoms within such confined spaces further and substantially reduces the desolvation energy, thus synergistically accelerating ion desolvation and boosting capacitive performance. Finite element analysis indicates hierarchical porous structure is more conducive to improving the zinc-ion storage kinetics. Leveraging the optimized HHPC-2 as the cathode, the resultant ZICs exhibit superior specific capacitance, exceptional cycling stability exceeding 50,000 cycles, and a high energy density of 120.0 Wh kg^−1^. Owing to its straightforward process, low‑cost reagents, high yield, and gram‑scale preparation, the dual-molten‑salt method presented here offers a scalable and practical route to produce high‑performance carbon materials for ZICs. Notably, this material enables air-mediated self-charging post-full discharge, achieving 80.5% efficiency and a rate of 15 mAh g^−1^ h^−1^, while integration with a Zn(CF_3_SO_3_)_2_/PVA gel electrolyte yields flexible ZICs poised for practical deployment in wearable and off-grid applications.

## Experimental Section

Pyrrole (AR, Macklin), methyl orange (MO, Shanghai Macklin Biochemical Co., Ltd.), anhydrous iron chloride (FeCl_3_, 98%, Beijing InnoChem Science & Technology Co., Ltd.), anhydrous zinc chloride (ZnCl_2_, 98%, Aladdin), zinc sulfate heptahydrate (ZnSO_4_, 99.5%, Macklin), polyvinylidene fluoride (PVDF, Shanghai Dibai Biotechnology Co., Ltd.), N-methyl-2-pyrrolidone (99%, Tianjin Fuchen Chemical Reagents Factory), anhydrous ethanol (AR, Tianjin Da Mao Chemical Reagent Co., Ltd.), concentrated hydrochloric acid (AR, Tianjin Kermel Chemical Reagent Co., Ltd.), and polyvinyl alcohol (PVA, Macklin) were used as received without further purification.

## Results and Discussion

### Pore Structure and Heteroatom Analysis

The synthesis process of HHPC-*x* is illustrated in Fig. 1b. Initially, methyl orange (MO) and FeCl_3_ form multifunctional self-assembled fibrous templates. Subsequently, the introduction of pyrrole monomers, leveraging the high reactivity of their α-H and planar conjugated molecular structure, initiates rapid polymerization under the oxidation of Fe^3+^. This process, templated by the MO-FeCl_3_ complex, proceeds via an in situ oxidative polymerization mechanism, leading to the directional growth of highly ordered one-dimensional (1D) polypyrrole nanotubes (PPyNTs). To further enhance the specific surface area (SSA) and the porous structure, a dual-molten-salt system (ZnCl_2_ and FeCl_3_) is employed as an activation agent. Our previous research indicated that ZnCl_2_ promoted the formation of abundant micropores and a sheet-like morphology, whereas FeCl_3_ helps preserve heteroatom content and graphitic structure [33]. With the mass ratio of PPyNT to ZnCl_2_ fixed, the amount of FeCl_3_ is varied to investigate its influence on the resulting porous structure and heteroatom configuration. The obtained samples are denoted as HHPC-*x*, where *x* represents the mass ratio of ZnCl_2_ to FeCl_3_.

**Fig. 2:**
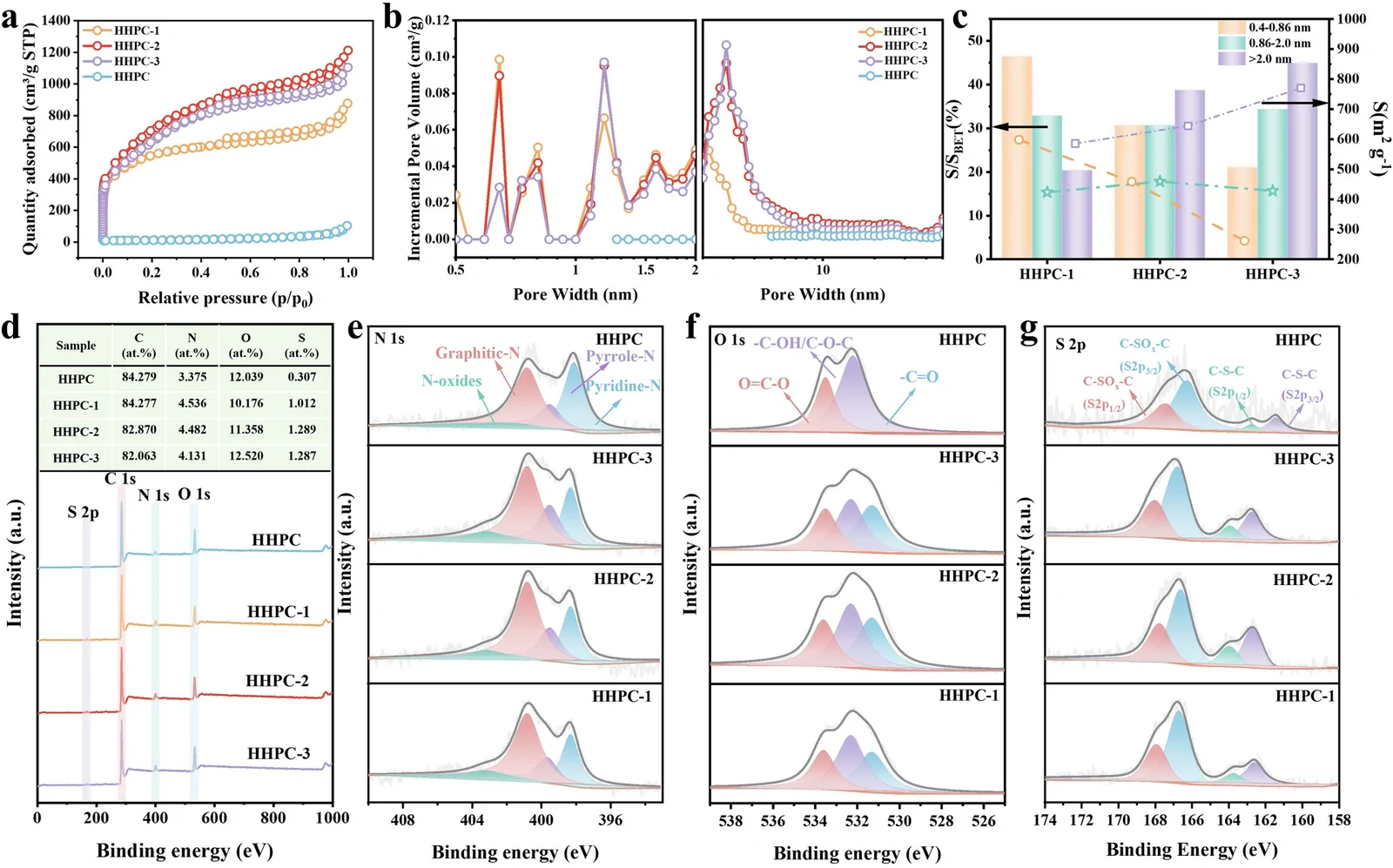
Pore structure and heteroatom analysis of HHPC and HHPC-X. **a** N_2_ adsorption/desorption isotherms. **b** Pore size distribution. **c** Proportion of SSA across different pore size ranges. **d** XPS survey spectra and high-resolution XPS spectra of **e** N 1*s*, **f** O 1*s*, and **g** S 2*p*

The SSA and pore structure of the materials are analyzed using nitrogen adsorption–desorption isotherms. As shown in Fig. 2a, all HHPC-*x* samples exhibit typical type-IV isotherms, characterized by a sharp uptake at low relative pressures (*P*/*P*_0_ < 0.01) and distinct H4-type hysteresis loops in the *P*/*P*_0_ range of 0.5–0.95, indicating the presence of hierarchical porosity comprising micropores, mesopores, and macropores [34]. Notably, the SSA of the molten-salt-treated HHPC-*x* samples is significantly higher than that of the untreated HHPC (43.6 m^2^ g^−1^). As the amount of molten FeCl_3_ increases, the SSA is enhanced from 1810.0 m^2^ g^−1^ (HHPC-1) to 2523.0 m^2^ g^−1^ (HHPC-2), while a further increase in FeCl_3_ results in a comparable SSA for HHPC-3 (2325.3 m^2^ g^−1^). A high SSA typically exposes more heteroatom sites, providing abundant active sites for ion adsorption and thereby enhancing electrochemical performance [35]. Interestingly, a more detailed analysis of the pore size distribution (PSD) plots (Fig. 2b) reveals that with increasing FeCl_3_ content, the proportion of ultramicropores decreases significantly from 46.4% in HHPC-1 to 21.0% in HHPC-3, while the mesopore content increases markedly from 20.4% to 45.0% (Fig. 2c and details can be seen in Table S1). This can be attributed to the fact that ZnCl_2_ facilitates the formation of micropores, while FeCl_3_ contributes to the construction of mesopores during the pyrolysis process. It is commonly assumed that ultramicropores (< 0.86 nm, the size of [Zn(H_2_O)_6_]^2+^) are ineffective in ZICs due to size exclusion [34, 36]. Intriguingly, the ultramicropore fraction in our synthesized samples approaches 50%, motivating a deeper investigation into the role of these traditionally “inactive” pores in Zn^2+^ storage using this material platform.

The surface functional groups and elemental composition/bonding of the samples are characterized by Fourier transform infrared (FTIR) spectroscopy and X-ray photoelectron spectroscopy (XPS), respectively. The FTIR spectra (Fig. S1) show a broad peak at 3000–3500 cm^−1^, corresponding to N–H stretching vibrations. The peaks near 1582 and 1468 cm^−1^ are attributed to the pyrrole ring skeleton vibrations, and the absorption peak near 1115 cm^−1^ is assigned to C-N stretching vibrations. The peak centered at 797 cm^−1^ represents N–H bending vibrations [37]. Moreover, the peaks at 1038 and 665 cm^−1^ are characteristic of sulfone (S=O) and C–S bonds, respectively, confirming the successful incorporation of N and S atoms into the carbon matrix.

XPS is used to analyze the surface chemical composition. The survey spectra (Fig. 2d) show that the S content increases from 0.307 at% in HHPC to 1.289 at% in HHPC-2 after dual-molten-salt treatment. Details for the deconvolution of C 1*s* (Fig. S2), N 1*s*, O 1*s*, and S 2*p* (Fig. 2e–g) spectra can be seen in the supporting information. Quantitative analysis (inset of Fig. 2d and Tables S2–S3) reveals a significant increase in the –C=O ratio in HHPC-*x* compared to HHPC, indicating that anhydrous FeCl_3_ aids in retaining oxygen content and promotes the formation of –C=O groups during pyrolysis, consistent with our previous reports [33]. Since –C=O groups are recognized as crucial sites for reversible Zn^2+^ storage, this suggests that HHPC-*x* materials are more suitable for ZIC cathodes. Comparing the XPS results for O, N, and S elements among HHPC-1, HHPC-2, and HHPC-3 (Tables S2 and S3) shows that the three samples exhibit nearly identical surface chemistry and heteroatom configurations, implying that varying the FeCl_3_ amount would not significantly alter the heteroatom content or bonding states. This consistency ensures that the heteroatom influence remains constant, allowing us to utilize this material series to isolate and study the sole effect of pore structure on Zn^2+^ storage performance.

### Microstructural Characterization

**Fig. 3:**
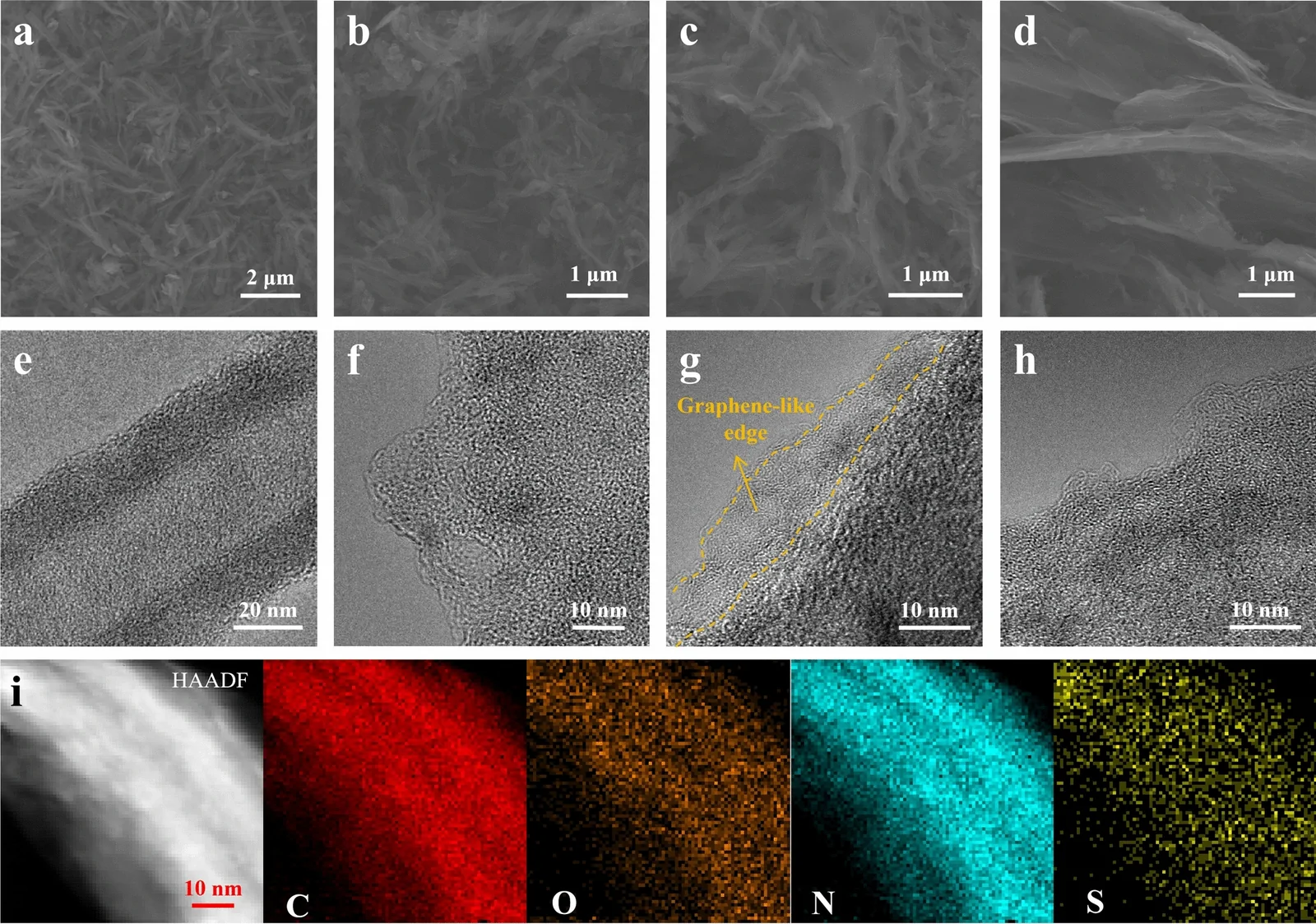
Microscopic morphology analysis of HHPC and HHPC-*x*: SEM images of **a** HHPC; **b** HHPC-1; **c** HHPC-2; **d** HHPC-3. TEM images of **e** HHPC; **f** HHPC-1; **g** HHPC-2; **h** HHPC-3. **i** HAADF-STEM image and corresponding elemental mapping of HHPC-2

The morphology evolution of HHPC and HHPC-x is first investigated by scanning electron microscopy (SEM). As shown in Fig. 3a, HHPC exhibits a typical 1D hollow carbon nanotube morphology. After treatment with the mild FeCl_3_/ZnCl_2_ molten salt, HHPC-1 largely retains the 1D mesoporous nanotube structure (Fig. 3b). Increasing the FeCl_3_ content results in HHPC-2, which shows a coexisting morphology of both nanotubes and nanosheets (Fig. 3c). A further increase in FeCl_3_ leads to HHPC-3, which primarily presents a nanosheet morphology (Fig. 3d). This morphology evolution is likely due to the exfoliation of the nanotube surfaces caused by the more intense reaction and high-temperature carbonization with increasing FeCl_3_ content. Further analysis by high-resolution transmission electron microscopy (HRTEM, Fig. 3e–h) confirms that all molten-salt-treated samples display features of amorphous carbon with disordered nanopores alongside partially graphitized microcrystalline domains. This phenomenon stems from the synergistic effect of the two salts: ZnCl_2_ favors the formation of porous amorphous carbon, while FeCl_3_ facilitates the ordered reconstruction of disordered structures and the formation of graphitic microcrystals [38]. Interestingly, HHPC-2 features ultrathin and graphene-like edges on its surface (Fig. 3g), which are beneficial for rapid electron transfer [39]. Furthermore, elemental mapping (Fig. 3i) of HHPC-2 confirms the uniform distribution of O, N, and S elements within the carbon framework.

X-ray diffraction (XRD) is employed to determine the crystal structure (Fig. S3). All HHPC-x samples show broad diffraction peaks at ~ 25.6° and ~ 43.6°, corresponding to the (002) and (100) planes of graphitic carbon [35], respectively. Compared to HHPC, the molten-salt-treated samples exhibit significantly broadened peaks, indicating increased structural defects and disordered regions [40], leading to lower crystallinity and enhanced amorphous characteristics, consistent with the HRTEM observations.

Raman spectroscopy is used to systematically study the defect structures (Fig. S4). The spectra are fitted into four peaks: *D*_2_ (~ 1250 cm^−1^, attributed to *sp*^3^ carbon or graphene edge defects), *D* (~ 1352 cm^−1^, related to lattice defects/disorder), *D*_1_ (~ 1468 cm^−1^, associated with amorphous carbon structures), and *G* (~ 1582 cm^−1^, representing the in-plane vibration of *sp*^2^-hybridized carbon) [41]. The distinct 2D band (~ 2700 cm^−1^) observed in all samples suggests the presence of ordered graphene-like layers [42]. The intensity ratio of the D to G bands (I_D_/I_G_) is commonly used to evaluate the defect degree [43]. As shown in Fig. S5, the HHPC-*x* samples show significantly higher I_D_/I_G_ values compared to HHPC (0.90), confirming an increased defect density after molten-salt treatment. This increase is attributed to the more developed porous structure and the preservation of S heteroatoms, as S-doping tends to occur at edge sites and defects, introducing local lattice strain and topological defects [44]. Consequently, the enriched defect structures in HHPC-*x* likely provide additional active sites for Zn^2+^ adsorption/desorption, enhancing the specific capacitance. Notably, the I_D_/I_G_ values for the HHPC-*x* series are very close (0.99 for HHPC-1, 1.03 for HHPC-2, and 1.01 for HHPC-3), indicating a comparable defect level across these samples. This allows for the investigation of pore structure effects on Zn^2+^ storage performance while effectively excluding the variable of defect density.

### Electrochemical Performance Investigation

The comprehensive structural analysis confirms that the HHPC-*x* series exhibits significant differences primarily in their pore architectures, while maintaining comparable heteroatom content and defect density. This unique attribute establishes an ideal platform for investigating the structure–performance relationship between pore structure and Zn^2+^ storage performance. Consequently, a detailed evaluation of their electrochemical behavior is conducted to elucidate the critical role of pore structure.

**Fig. 4:**
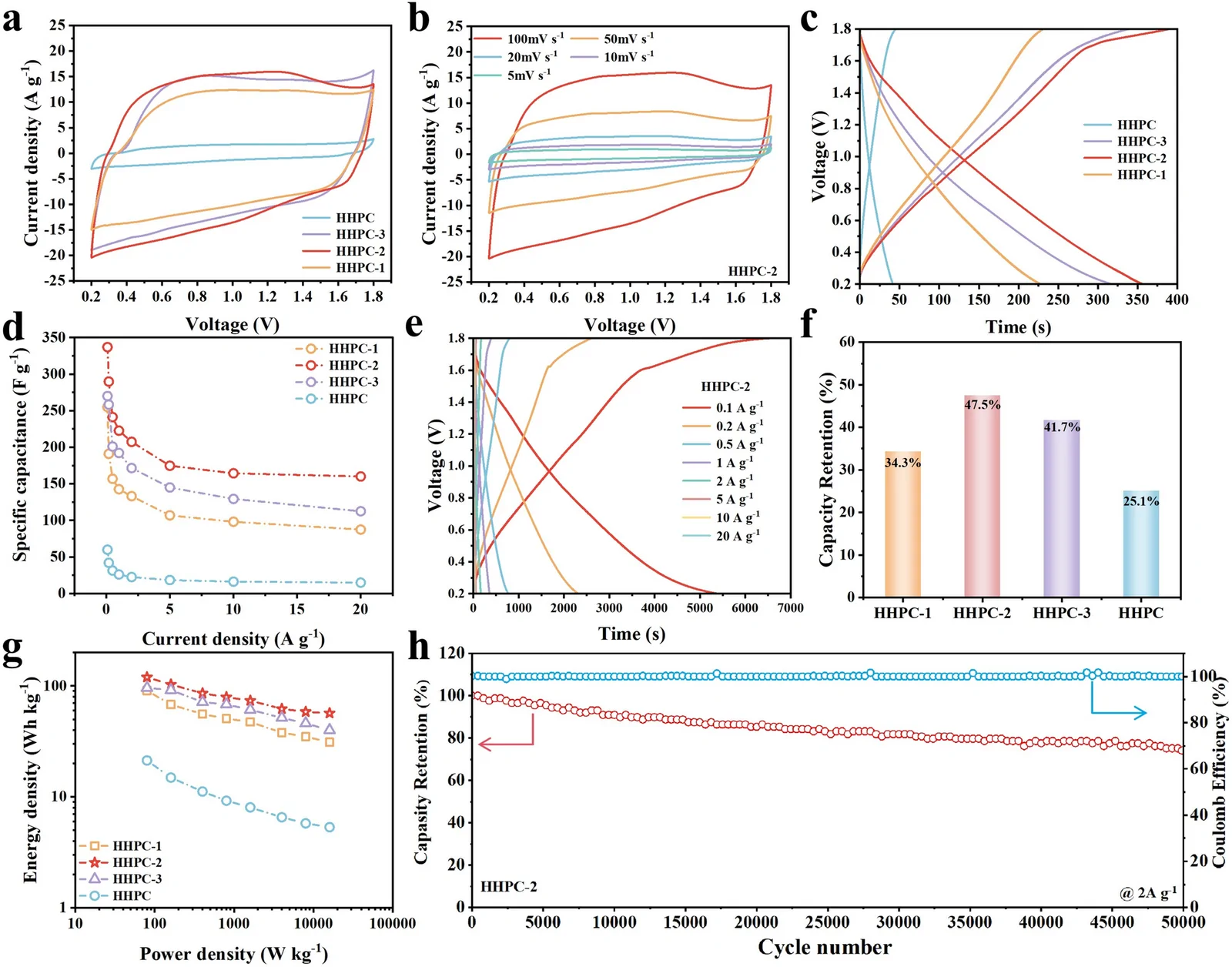
Electrochemical performance evaluation of ZICs. **a** CV curves at a scan rate of 100 mV s^−1^. **b** CV curves of HHPC-2 at different scan rates. **c** GCD profiles at a current density of 1 A g^−1^. **d** Rate capability at various current densities. **e** GCD profiles of HHPC-2 under different current densities. **f** Capacity retention and **g** comparison of Ragone plots for HHPC-x based ZICs. **h** Cycling performance of HHPC-2 at 2 A g^−1^

ZICs are assembled by pairing different carbon materials as cathodes with zinc foil anodes. Cyclic voltammetry (CV) curves at 100 mV s^−1^ (Fig. 4a) display a quasi-rectangular shape coupled with a pair of broad redox peaks, indicating a combination of dominant electric double-layer capacitive (EDLC) behavior and reversible faradaic processes. The redox peaks are attributed to Zn/Zn^2+^ plating/stripping and the chemisorption of Zn^2+^/H^+^ facilitated by oxygen-containing functional groups [45]. HHPC-2 exhibits the largest CV integrated area, suggesting the highest specific capacitance. Furthermore, CV curves at various scan rates (5–100 mV s^−1^, Figs. 4b and S6) maintain quasi-rectangular shapes for HHPC-*x* samples, whereas HHPC shows severe polarization, underscoring the enhanced charge transfer kinetics in the molten-salt-treated materials. As a result, it can be deduced that the improvement is attributed to the well-tailored hierarchical pore structure, which facilitate charge carrier transport.

Galvanostatic charge–discharge (GCD) profiles at 1 A g^−1^ (Fig. 4c) further corroborate these findings, with HHPC-2 delivering the highest specific capacitance of 222.6 F g^−1^, outperforming HHPC-1 (142.6 F g^−1^), HHPC-3 (192.1 F g^−1^), and HHPC (26.0 F g^−1^). GCD tests at different current densities (Figs. 4d, e and S7) reveal that HHPC-2 achieves a superior specific capacitance of 336.9 F g^−1^ at 0.1 A g^−1^. Notably, it retains 160.0 F g^−1^ (47.5% retention) even at a high current density of 20 A g^−1^, significantly exceeding HHPC-1 (34.3%) and HHPC-3 (41.7%), demonstrating excellent rate performance and reversibility (Fig. 4f and Table S4). The Ragone plot indicates the HHPC-2-based ZIC delivers a high energy density of 120.0 Wh kg^−1^ at a power density of 80 W kg^−1^, which is superior to the other HHPC-based ZICs (Fig. 4g). Moreover, the Zn^2+^ storage capacitance of HHPC-2 ranks among the highest reported for carbon-based cathodes (Fig. S8 and Table S5). Additionally, the HHPC-2-based ZIC exhibits exceptional long-term cycling stability, maintaining 74.1% of its initial capacitance after 50,000 cycles with nearly 100% Coulombic efficiency (Figs. 4h and S9), underscoring its great promise for practical ZICs.

### Elucidating the Ultramicropore Effect (UME) and Hierarchical Porous Effect (HPE)

Two key observations from the electrochemical performance warrant further investigation: (1) HHPC-1 and HHPC-3, despite their large difference in SSA (1810.0 vs. 2325.3 m^2^ g^−1^) but similar heteroatom/defect levels, exhibit nearly identical capacitances at low current densities (255.0 vs. 270.0 F g^−1^); (2) HHPC-3 possesses a higher mesoporous surface area than HHPC-2 (770.5 vs. 644.5 m^2^ g^−1^, Table S1), yet HHPC-2 demonstrates a superior rate capability (capacity retention of 47.5% vs. 41.7%).

**Fig. 5:**
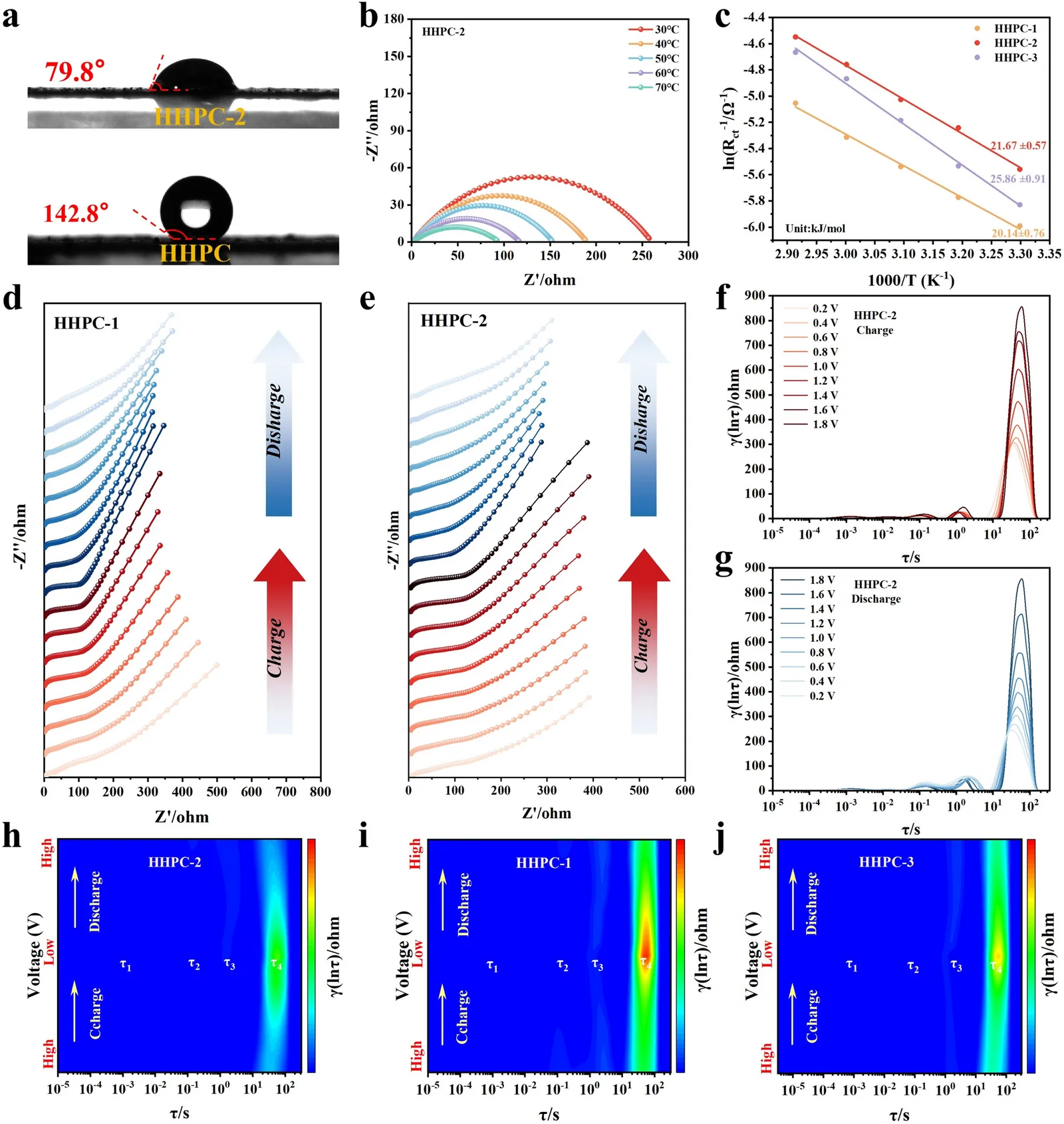
**a** Contact angle test of HHPC and HHPC-2. **b** EIS spectra at different temperatures for Zn//Zn symmetric cells with HHPC-2 and **c** corresponding calculated Ea values; in situ EIS plots of **d** HHPC-1//Zn and **e** HHPC-2//Zn; DRT analysis derived from in situ EIS measurements of HHPC-2//Zn during **f** charging and **g** discharging process; in situ DRT plots of ZICs based on **h** HHPC-2, **i** HHPC-1, and **j** HHPC-3

Contact angle measurements and desolvation behavior analysis are employed to probe the UME. The contact angle decreases dramatically from 142.8° for HHPC to 85.4°, 79.8°, and 59.4° for HHPC-1, HHPC-2, and HHPC-3, respectively (Figs. 5a and S10), indicating significantly improved hydrophilicity after molten-salt treatment. Given that the primary change in surface functional groups is the introduction of S, the enhanced wettability is largely attributed to S-doping, which facilitates electrolyte accessibility and reduces electrode/electrolyte interface impedance. However, among HHPC-*x* samples with comparable heteroatom content, the gradual decrease in contact angle suggests that pore structure also critically influences wettability. Moderate hydrophobicity is believed to promote the desolvation of [Zn(H_2_O)_6_]^2+^ by repelling water molecules from the solvation sheath [25], thereby lowering the energy barrier for Zn^2+^ migration. Thus, the gradually increased hydrophilicity from HHPC-1 to HHPC-3 implies a weakening desolvation capability, a trend that correlates with the decreasing proportion of ultramicropores (Fig. 2c). This links the UME primarily to the identical capacitances at low current densities between HHPC-1 and HHPC-3.

To quantitatively assess the desolvation kinetics, the activation energy (*E*_a_) for the desolvation of [Zn(H_2_O)_6_]^2+^ is determined by constructing Zn//Zn symmetric cells with HHPC-*x* as an interfacial layer and analyzing temperature-dependent electrochemical impedance spectroscopy (EIS, Figs. 5b and S11) using the Arrhenius equation [14]. The HHPC-1 modified electrode consistently exhibits the lowest charge transfer resistance, indicating its superior capability to facilitate desolvation. Quantitative analysis yields *E*_a_ values of 20.14, 21.67, and 25.86 kJ mol^−1^ for HHPC-1, HHPC-2, and HHPC-3, respectively (Fig. 5c), conclusively demonstrating that the abundant ultramicropores in HHPC-1 significantly lower the desolvation energy barrier. These results unequivocally establish the critical role of the UME in enhancing Zn^2+^ storage capacity by promoting desolvation, explaining why HHPC-1 and HHPC-3, despite their SSA difference, deliver comparable capacitances at low current densities.

In situ EIS analysis is conducted to unravel the influence of HPE on the charge transport kinetics during ZIC operation. The charge transfer resistance (*R*_ct_) in the high-frequency region increases significantly near 0.2 V but decreases markedly at 1.8 V (Figs. 5d, e and S12), primarily due to the precipitation and dissolution of Zn_4_SO_4_(OH)_6_·5H_2_O. Distribution of relaxation times (DRT) method of the in situ EIS data acquired during GCD at 0.5 A g^−1^ (Figs. 5f, g and S13) deconvolutes the processes into four characteristic time constants [46]: τ_1_ (~ 10^–3^ s, related to internal electrode contact resistance), τ_2_ (~ 10^–1^ s, associated with interfacial charge transfer), τ_3_ (~ 1 − 10 s, assigned to short-range carrier diffusion within the electrode bulk), and τ_4_ (> 10 s, corresponding to long-range diffusion across the cell). The *R*_ct_ for HHPC-2//Zn decreases initially and then increases during discharge, attributable to the influence of by-products formed at low potentials on mass transport (Fig. 5h). Crucially, the τ_2_, τ_3_, and τ_4_ signals for HHPC-2 exhibit the fastest attenuation, indicating the most rapid interfacial reaction kinetics and charge diffusion rates. In contrast, HHPC-1 shows the poorest diffusion kinetics (Fig. 5i), while HHPC-3, with more mesopores, performs better than HHPC-1 but worse than HHPC-2 (Fig. 5j). This confirms that the HPE predominantly governs the ion transport kinetics, explaining why HHPC-2, with its optimal hierarchical pore distribution, outperforms HHPC-3 in rate capability, despite their similar SSAs.

### Theoretical Simulations on the UME and HPE

To gain deeper mechanistic insights into the observed enhancement in Zn^2+^ storage kinetics, theoretical simulations are employed to decouple the individual and synergistic effects of pore architecture and heteroatom doping on the desolvation and transport processes of hydrated zinc ions.

**Fig. 6:**
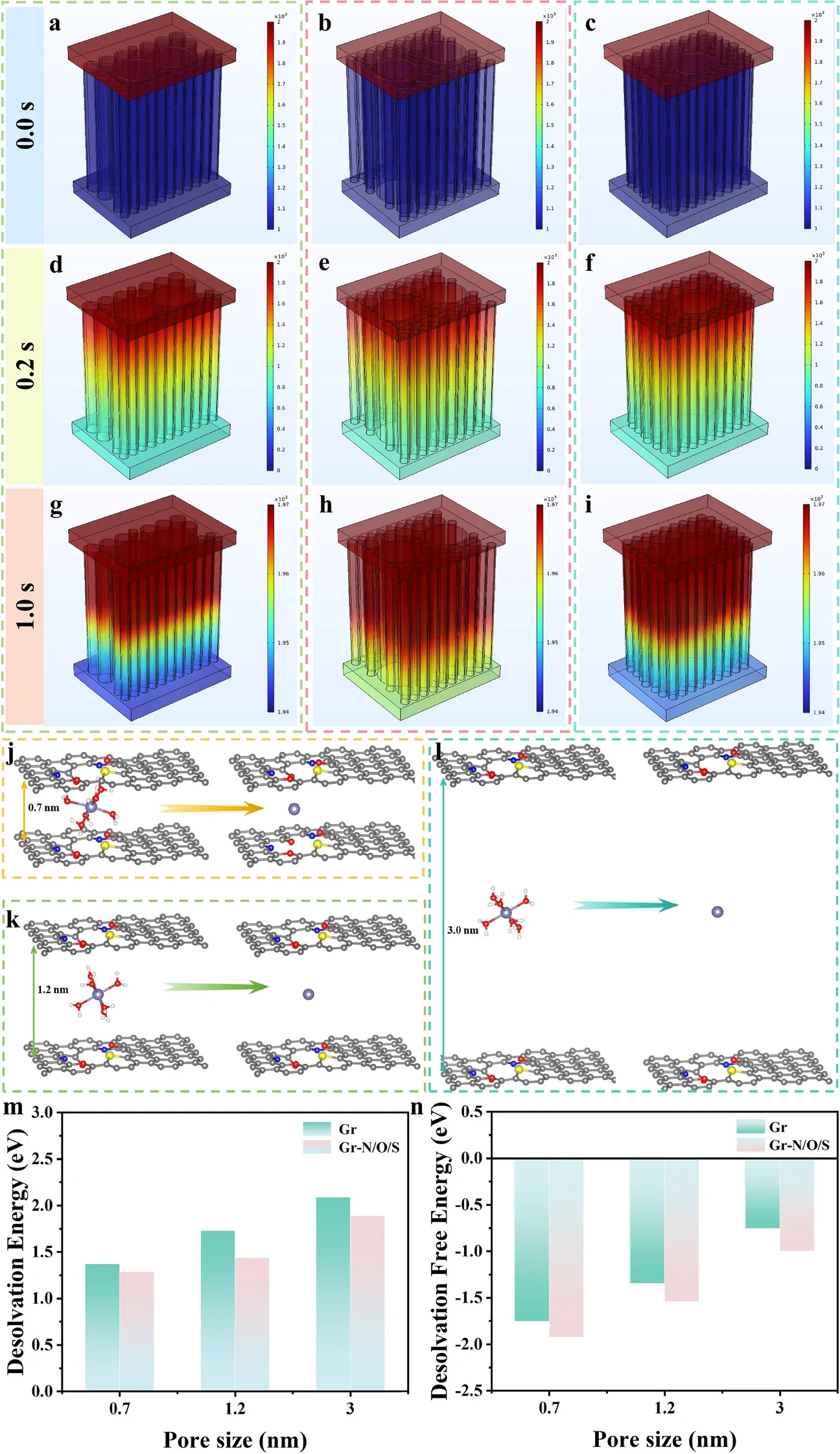
Theoretical analysis for the UME and HPE. [Zn(H_2_O)_6_]^2+^ distribution by the finite element simulation for **a**, **d**, **g** Model-1, **b**, **e**, **h** Model-2, and **c**, **f**, **i** Model-3 at different times; the model structures for the desolvation of [Zn(H_2_O)_6_]^2+^ ions based on double-layer N/O/S-doped graphene with layer distances of **j** 0.7 nm, **k** 1.2 nm, and **l** 3.0 nm. **m** Desolvation energy and **n** desolvation free energy of [Zn(H_2_O)_6_]^2+^ on all the model structures

Finite element simulations are first performed using COMSOL calculations to simulate the diffusion and permeation of [Zn(H_2_O)_6_]^2+^, through carbon pore networks with structural characteristics analogous to the HHPC-x series. Three distinct models are constructed: Model-1 (Fig. 6a), featuring a high ultramicropore (UME, < 0.86 nm) fraction (46.4%); Model-3 (Fig. 6c), dominated by a high mesopore fraction (45.0%); and Model-2 (Fig. 6b), characterized by a uniform and balanced distribution of ultramicropores, micropores, and mesopores. The total micropore volume is kept consistent (~ 33%) across all models. The simulation results monitoring ion transport over a 1-s relaxation period reveal that diffusion and permeation are incomplete in both Model-1 (Fig. 6d, g) and Model-3 (Fig. 6f, i). In contrast, Model-2 demonstrates significantly more facile and virtually complete diffusion of the [Zn(H_2_O)_6_]^2+^ ions (Fig. 6e, h). This finding provides numerical evidence for the HPE, confirming that a uniformly distributed hierarchical pore network is most conducive to the rapid transport of solvated Zn^2+^, thereby facilitating overall ionic flux.

DFT calculations are further conducted to quantify the energy barrier associated with the desolvation of [Zn(H_2_O)_6_]^2+^ within confined pore spaces. The calculation strategy involves two sets of bilayer graphene slit-pore models reflecting the characteristic pore sizes. The first set (Fig. S14) consists of pure carbon models with slit widths of 0.7 nm (Gr-1), 1.2 nm (Gr-2), and 3.0 nm (Gr-3). The second set (Fig. 6j, k and l), Gr-N/O/S-1, Gr-N/O/S-2, and Gr-N/O/S-3, is derived from the first set by incorporating identical amounts of N, O, and S heteroatoms into the carbon lattice.

The calculations focus on the desolvation energy (*E*_d_) and Gibbs free energy change (*ΔG*_*d*_) for the complete desolvation of [Zn(H_2_O)_6_]^2+^ to a bare Zn^2+^ ion within these pore models. Analysis of the pristine carbon models (Gr-1, Gr-2, and Gr-3) reveals relatively high energy barriers (Fig. 6m, n) for desolvation compared with the models with heteroatoms. Notably, the process is particularly unfavorable in the 3.0 nm mesopore (Gr-3), suggesting significant steric and energetic constraints. A critical finding emerges upon introducing heteroatoms: The desolvation energy barrier in the N/O/S-doped ultramicropore (Gr-N/O/S-1) is dramatically reduced (Fig. 6m, n) and proves to be the lowest (in terms of both *E*_d_ and *ΔG*_*d*_) among all models. This result unequivocally demonstrates that the UME is significantly enhanced by the presence of heteroatoms. The synergistic combination of ultramicropore confinement and favorable surface chemistry created by heteroatom doping effectively lowers the energy barrier for stripping water molecules from the Zn^2+^ solvation shell.

In sum, the theoretical calculations, in combination with the experimental results, provide a coherent theoretical framework: The HPE ensures efficient macroscopic [Zn(H_2_O)_6_]^2+^ transport through a well-interconnected pore network, while the heteroatom-enhanced UME governs the localized microscopic desolvation kinetics at the subnanometer scale. Their synergistic cooperation is fundamental to the superior electrochemical performance observed in the optimally designed HHPC-2 material.

### Charge Storage Mechanism Analysis

**Fig. 7:**
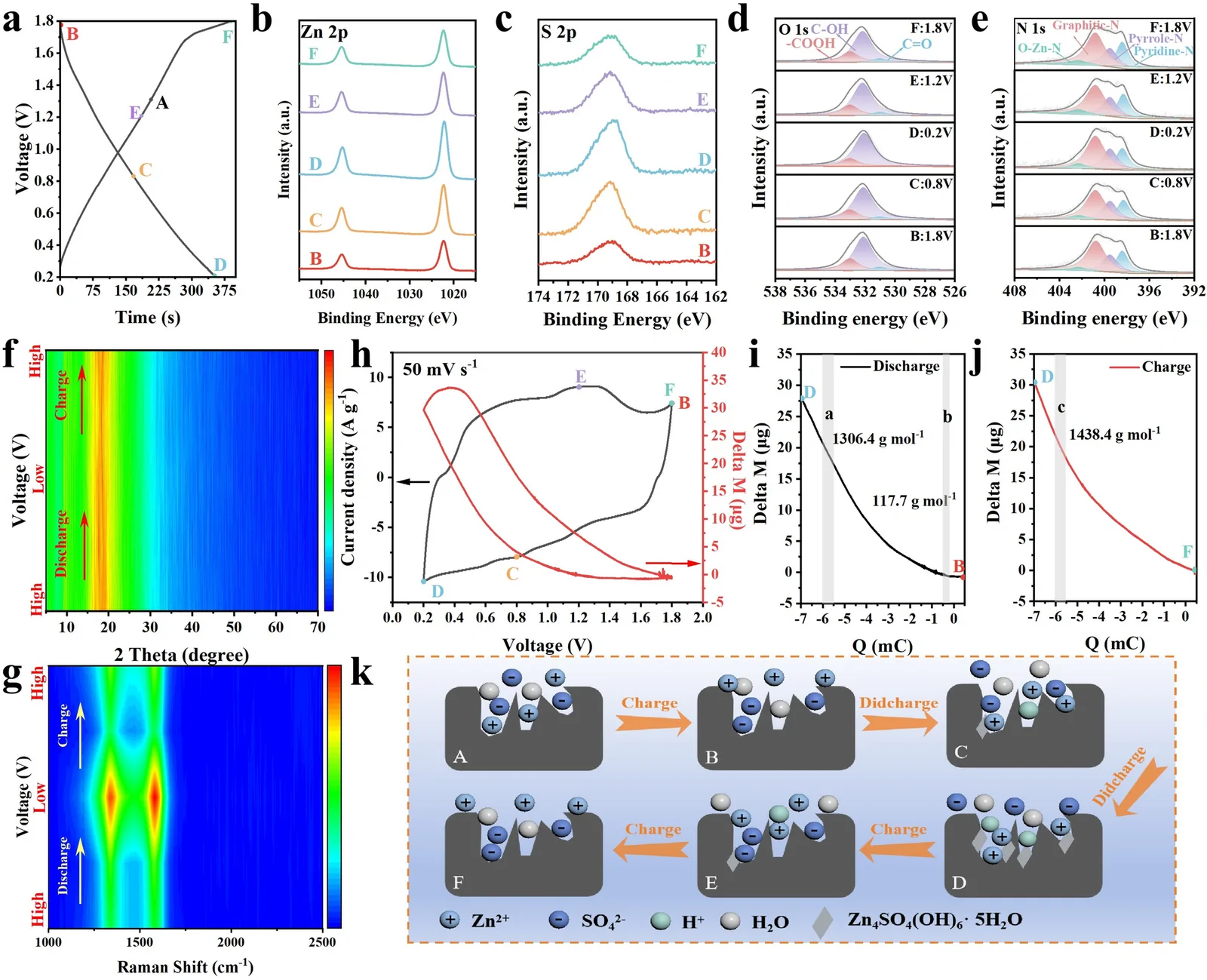
**a** Galvanostatic charge/discharge profile of HHPC-2. Ex situ XPS spectra of the HHPC-2 cathode during the charge/discharge process: **b** Zn 2*p*; **c** S 2*p*; **d** O 1*s*; **e** N 1*s*; **f** Ex situ XRD patterns. **g** Ex situ Raman spectra.** h** EQCM measurement of the HHPC-2 cathode at 50 mV s^−1^. Mass variation versus charge curves during **i** discharge and **j** charge processes. **k** Schematic diagram of the charge storage mechanism in the HHPC-2 cathode

To gain deeper insights into the charge storage mechanism, a series of ex situ characterizations (XPS, Raman and XRD) are performed on the HHPC-2 cathode at different states of charge/discharge (Fig. 7a): pristine (State A), during discharge to 0.8 V (C) and 0.2 V (D), and during charge to 1.2 V (E) and 1.8 V (F).

Ex situ XPS is used to track chemical composition evolution (Fig. S15). The Zn 2*p* signal intensity (Fig. 7b) increases during discharge due to Zn^2+^ adsorption and decreases during charging as Zn^2+^ desorbs, confirming highly reversible Zn^2+^ adsorption/desorption. Concurrently, the S 2*p* signal (Fig. 7c), associated with SO_4_^2−^ adsorption and Zn_4_SO_4_(OH)_6_·5H_2_O, intensifies during discharging process, peaks at the fully discharged state (D), and weakens during charging, indicating the reversible deposition/dissolution of Zn_4_SO_4_(OH)_6_·5H_2_O [47]. This by-product forms at low potentials due to a localized pH increase from H^+^ adsorption and dissolves at high potentials.

Further analysis of the O 1*s* and N 1*s* high-resolution XPS spectra (Fig. 7d, e) reveals reversible changes. The C=O signal intensity decreases from State B (1.8 V) to State D (0.2 V) and recovers upon charging to State F (1.8 V), while the C–OH signal shows the opposite trend. This demonstrates that C=O groups participate in reversible redox reactions with Zn^2+^, enhancing chemisorption. The N 1*s* spectra show a reversible decrease in pyridinic-N signal during discharge and recovery during charge, accompanied by the emergence and disappearance of an O–Zn–N peak at 402.3 eV, confirming that pyridinic-N sites also contribute to reversible Zn^2+^ chemisorption.

Ex situ XRD patterns (Fig. 7f) show that the (002) diffraction peak remains stable throughout the cycling without significant intensity changes, indicating excellent structural stability of HHPC-2 and suggesting that Zn^2+^ storage primarily involves reversible adsorption/desorption, with capacitance mainly contributed by EDLC. Ex situ Raman spectra (Fig. 7g) show an increased defect level (I_D_/I_G_ ratio) during discharge, resulting from Zn^2+^/H^+^ adsorption, which reverses upon charging, again confirming the highly reversible charge adsorption/desorption.

Electrochemical quartz crystal microbalance (EQCM) is employed for real-time mass change analysis during CV cycling (Fig. 7h). The molar mass (M/z) of the adsorbed/desorbed species and the solvation number are calculated based on the EQCM measurement. In region a (Fig. 7i), the large M/z value of 1306.4 g mol^−1^ corresponds to the adsorption of 2 Zn_4_SO_4_(OH)_6_·5H_2_O precipitates, 12 H^+^, and 3 Zn^2+^. Region b (M/z = 117.7 g mol^−1^) involves the adsorption of 5.5 Zn^2+^ and desorption of 2.5 SO_4_^2−^. During charging, region c (Fig. 7j, M/*z* = 1438.4 g mol^−1^) corresponds to the dissolution of 2 Zn_4_SO_4_(OH)_6_·5H_2_O, desorption of 14 H^+^, and 5 Zn^2+^. The EQCM results conclusively demonstrate that the charge storage mechanism involves the co-adsorption/desorption of Zn^2+^, H^+^, and SO_4_^2−^, coupled with the reversible precipitation/dissolution of Zn_4_SO_4_(OH)_6_·5H_2_O.

Based on the combined ex situ and in situ analyses, the charge storage mechanism for the HHPC-2-based ZIC is summarized in Fig. 7k: (1) co-adsorption of Zn^2+^/H^+^ (low-voltage region) and SO_4_^2−^ (high-voltage region), (2) reversible formation/dissolution of Zn_4_SO_4_(OH)_6_·5H_2_O, related to H^+^ participation, and (3) enhanced chemisorption provided by N/O/S dopants, particularly C=O and pyridinic-N groups, which lower the adsorption energy barrier for Zn^2+^.

### Electrochemical Kinetics Analysis

In electrochemical impedance spectroscopy (EIS), the diameter of the semicircle in the high-frequency region of the Nyquist plot is proportional to the charge transfer resistance, while the slope in the low-frequency region is inversely related to the ion diffusion resistance (*σ*) in the electrolyte [48]. The corresponding Nyquist plots (Fig. S16) reveal that the ZICs based on HHPC-2 exhibit the smallest internal resistance (*R*_e_) and charge transfer resistance (*R*_ct_), indicating superior charge transfer kinetics and excellent electrode/electrolyte interface compatibility of the HHPC-2 cathode material.

Furthermore, the relationship between phase angle and frequency for the HHPC-x cathodes (Fig. S17a) allows for the calculation of the relaxation time constant *τ*_0_ (*τ*_0_ = 1/*f*_0_) [11], where *f*_0_ is the characteristic frequency at a phase angle of − 45°. The calculated τ_0_ values for HHPC, HHPC-1, HHPC-2, and HHPC-3 are 84.0, 60.9, 42.1, and 54.3 s, respectively. Typically, a shorter relaxation time signifies faster charge transport kinetics [34]. Therefore, it is evident that HHPC-2 is most favorable for Zn^2+^ diffusion, which is associated with its more uniform hierarchical porous structure.

Additionally, the ion diffusion resistance (*σ*) is quantitatively analyzed by fitting the linear relationship between the real part of the impedance (*Z*′) and *ω*^−1/2^ (Fig. S17b). A lower *σ* value generally indicates faster ion migration during the electrochemical process [36]. The calculated results show that HHPC-2 possesses the lowest ion diffusion resistance (19.8 Ω s^−1/2^), significantly lower than that of HHPC (73.4 Ω s^−1/2^), HHPC-1 (47.0 Ω s^−1/2^), and HHPC-3 (39.7 Ω s^−1/2^). This confirms that HHPC-2 exhibits optimal Zn^2+^ diffusion capability. These kinetic parameter analyses are consistent with the pore size distribution results: HHPC-2, with its balanced abundance of micropores and mesopores, possesses an optimal hierarchical pore structure that effectively reduces ion transport resistance, shortens the Zn^2+^ diffusion path within the micropores, thereby lowering the diffusion energy barrier and significantly enhancing the Zn^2+^ transport rate.

To further investigate the energy storage mechanism of HHPC-*x*-based ZICs, the charge storage kinetics are analyzed using CV curves (Figs. 4b and S6) at different scan rates (from 5 to 100 mV s^−1^). The charge storage kinetics are studied based on the following equation:

1 $$i = av^{b}$$

where *a* and *b* are constants, *i* is the peak current, and *v* is the scan rate. The value of *b* (typically 0.5–1.0) is a key parameter for evaluating charge storage kinetics. A *b*-value of 0.5 indicates a diffusion-controlled process, while a *b*-value of 1.0 represents an ideal surface-capacitive-controlled process with fast kinetics [49]. The *b*-values for the cathodic and anodic peaks of HHPC-2 are 0.932 and 0.934, respectively, which are higher than other samples (Fig. S18), indicating that the charge storage is dominated by surface-capacitive behavior, accompanied by a partial diffusion-controlled contribution.

The Dunn’s method is employed to quantify the proportion of capacitive and diffusion-controlled contributions [49], using the following relationship between current (*i*) and scan rate (*v*):

2 $$i = k_{1} v + k_{2} v^{1/2}$$

3 $$\frac{i}{{v^{1/2} }} = k_{1} v^{1/2} + k_{2}$$

where *k*_1_ and *k*_2_ are constants. The *k*_1_*v* represents the current from the fast capacitive processes, while *k*_2_*v* represents the current from the slower diffusion-controlled processes. The analysis reveals that at a scan rate of 50 mV s^−1^, the charge storage mechanism of the HHPC-2 cathode is predominantly capacitive-controlled compared with HHPC and other HHPC-x samples (Fig. S19), with a contribution of 84.7%, while the diffusion-controlled contribution is 15.3%, demonstrating excellent rapid charge storage kinetics. Notably, as the scan rate increases from 5 to 100 mV s^−1^, the capacitive contribution ratio gradually increases from 71.6% to 91.7% (Fig. S20), which is obviously higher compared with other samples. This phenomenon reveals that at high scan rates, HHPC-2 exhibits surface-capacitive behavior driven by the fast kinetics of Zn^2+^/H^+^ (de)solvation and/or interfacial processes. The significantly higher contribution from fast kinetics further confirms the crucial role of the aforementioned UME and HPE in enhancing charge storage kinetics, resulting in high efficiency during charge storage and transfer.

### Practical Application Demonstration

#### Assembly of Flexible Zinc-Ion Capacitors

To demonstrate practical applicability, a flexible quasi-solid-state ZIC (FZIC) is assembled using HHPC-2 as the cathode, Zn foil as the anode, and a poly(vinyl alcohol)/Zn(CF_3_SO_3_)_2_ (PVA/ Zn(CF_3_SO_3_)_2_) gel electrolyte[50] (Fig. S21 and inset of Fig. S22). The flexible device exhibits excellent anti-self-discharge performance, maintaining a voltage of 1.29 V after 36 h (Fig. S22). CV (Fig. S23a) and GCD (Fig. S23b) curves within the voltage range of 0.2–1.8 V show typical capacitive characteristics. The FZIC delivers a specific capacitance of 100 F g^−1^ at 1 A g^−1^ and retains 58% of its capacitance at 10 A g^−1^ (Fig. S24), demonstrating reliable rate capability. Remarkably, the CV curves remain nearly identical under bending angles from 0° to 180° (Fig. S25a), highlighting outstanding mechanical flexibility and operational stability. Serial and parallel connections of two FZICs (Fig. S25b) successfully double the operating voltage and output current, respectively. The device achieves a maximum energy density of 41.64 Wh kg^−1^ (Fig. S26a) and maintains 88.5% capacitance after 7000 cycles (Fig. S26b). In addition, a single device powers a timer for over 300 h (Fig. S27a), and two serially connected devices illuminate five LED lights for 35 min (Fig. S27b), confirming its potential for wearable and portable electronics.

#### Construction of Air Self-charging ZICs

Intriguingly, HHPC-2 also exhibits excellent electrocatalytic activity for the oxygen reduction reaction (ORR) in ZnSO_4_ electrolyte, demonstrating a higher potential (1.20 V), onset potential (1.18 V) and limiting diffusion current density (2.18 mA cm^−2^) than HHPC-1 and HHPC-3 (Fig. S28). According to the linear sweep voltammetry (LSV) curves (Fig. S29a) in O_2_-saturated 1 M ZnSO_4_ on RRDE, the ORR process primarily follows a four-electron pathway (Fig. S29b). The superior ORR performance of HHPC-2 could be attributed to the coexistence of UME and HPE: The ultramicropores provide confinement for efficient O_2_ reduction, while the mesopores ensure rapid transport of ORR intermediates.

**Fig. 8:**
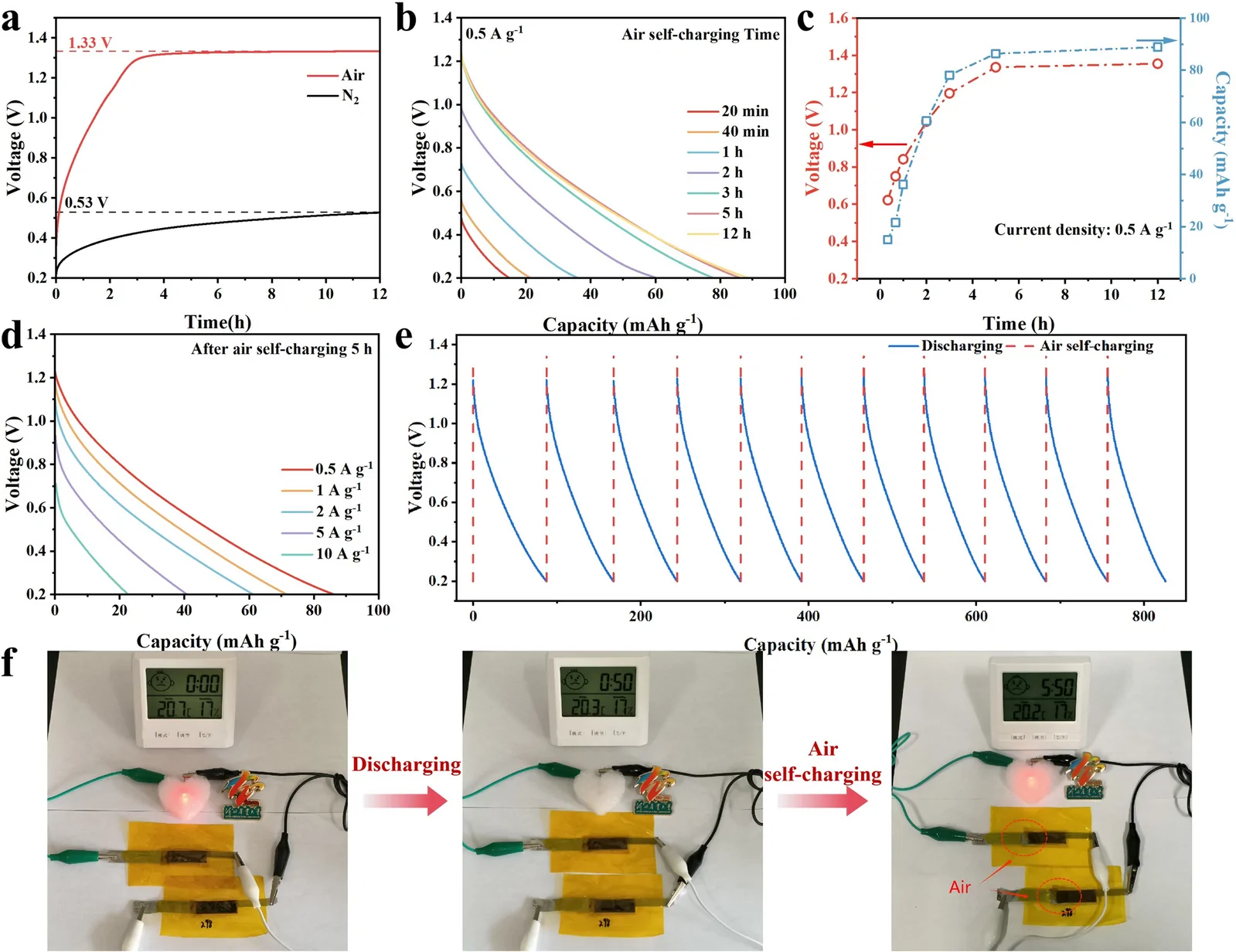
Electrochemical performance of the HHPC-2 cathode in air self-charging mode: **a** In situ air self-charging curves under air and N_2_ atmosphere. **b** Discharge curves within different self-charging times. **c** Relationship between discharging capacity and charging voltage with different self-charging times. **d** Rate capability of the HHPC-2 cathode after 5 h of air self-charging. **e** Self-charging/galvanostatic discharging cycling curves. **f** A red LED light powered by two flexible air self-charging HHPC-2-based SCZICs connected in series under different states

Leveraging this bifunctionality, i.e., Zn^2+^ storage and ORR catalysis, a self-charging ZIC (SCZIC) is constructed using HHPC-2 as the cathode. After being fully discharged, the HHPC-2 cathode can spontaneously be oxidized by environmental O_2_ via the ORR, achieving a self-charging process without an external power source. In an air atmosphere, the voltage of the device recovers to 1.33 V after 12 h (Fig. 8a). In contrast, in a N_2_ atmosphere, the self-charging voltage plateau is lower, confirming the crucial role of O_2_ participation in the cathode reaction. The discharge capacity after different air-charging durations is tested at 0.5 A g^−1^ (Fig. 8b, c). After 5 h of air charging, the device delivers a high specific capacity of 86.36 mAh g^−1^. Nonetheless, prolonging the air-charging time beyond 5 h can hardly further increase the capacity. The rate capability of the air-charging device for 5 h is shown in Fig. 8d, delivering capacities of 86.3, 71.7, 61.3, 40.8, and 22.5 mAh g^−1^ at 0.5, 1.0, 2.0, 5.0, and 10.0 A g^−1^, respectively. The SCZIC also demonstrates repeatable self-charging capability over 11 consecutive chemical charging (by air)/galvanostatic discharging cycles, with a cumulative capacity of 825.7 mAh g^−1^ at 0.5 A g^−1^ (Fig. 8e). The self-charging efficiency (*η*) and average self-charging rate (*ν*) reach 80.5% and 15 mAh g^−1^ h^−1^, respectively (details can be seen in the Supporting information). A practical demonstration is exhibited involving two flexible devices (Fig. 8f): After being fully discharged and the sealing film removed, exposure to air for 5 h allows them to recover enough charge to light a red LED again, validating the feasibility of this system as a reversible air-charging energy storage solution for use in remote or resource-limited environments.

## Conclusion

In this work, a molten-salt-assisted pyrolysis strategy, utilizing polypyrrole as the precursor, is developed to fabricate heteroatom-doped hierarchical porous carbon materials, featuring N/O/S co-doping synergized with ultramicroporous structures. By tuning the dual-molten-salt ratio, pore size distribution optimization and surficial chemistry modulation are realized. On the one hand, activation energy measurement coupled with DFT simulations reveals a dual enhancement mechanism: The spatial confinement imposed by 0.7 nm ultramicropores intrinsically facilitates the desolvation of [Zn(H_2_O)_6_]^2+^ compared to larger pores, and this ultramicropores effect (UME) is further amplified by the synergy with N/O/S heteroatoms, which significantly lowers the interaction energy barrier during the desolvation process, thereby substantially boosting the zinc-ion storage capacitance. On the other hand, in situ EIS test and finite element modulation disclose hierarchical porous effect (HPE) is more conducive to improving the zinc-ion storage kinetics. Structural characterizations confirm that the optimized HHPC-2 possesses a SSA of up to 2523 m^2^ g^−1^, with ultramicropores (< 0.86 nm) contributing 30.6% of the total SSA. Together with the high heteroatom contents, ZICs based on HHPC-2 demonstrate a high specific capacitance of 222.6 F g^−1^ at 1 A g^−1^, exceptional cycle stability (up to 50,000 cycles) and high energy density (120.0 Wh kg^−1^). Intriguingly, upon full discharge, exposing the cathode to air yields an air self-charging efficiency of 80.5% and a self-charging rate of 15 mAh g^−1^ h^−1^. Employing HHPC-2 as the electrode and a Zn(CF_3_SO_3_)_2_/PVA gel electrolyte, flexible zinc-ion capacitors are successfully constructed with robust mechanical resilience and electrochemical durability. Specifically, CV curves under bending angles from 0° to 180° remain virtually coincident, delivering a specific capacitance of 100 F g^−1^ at 1 A g^−1^ with a retention of 58% at 10 A g^−1^, underscoring their substantial potential for wearable and off-grid applications.

## Supplementary Information

Below is the link to the electronic supplementary material.

Supplementary file1 (DOCX 4829 KB)
