# Supplementary material for: Synergistic Ultramicropore and Hierarchical Pore Engineering in Heteroatom-Doped Carbon for High-Performance Zinc-Ion Capacitors
Source: Nanomicro Lett. 2026 Apr 17;18:336. doi: 10.1007/s40820-026-02181-0 (PMC13090442; doi:10.1007/s40820-026-02181-0)
Supplement: Supplementary file 1 — Supplementary file1 (DOCX 4829 KB) [file 40820_2026_2181_MOESM1_ESM.docx]

Supporting Information for

**Synergistic Ultramicropore and Hierarchical Pore Engineering in Heteroatom-Doped Carbon for High-Performance Zinc-Ion Capacitors**

Jiale Zhang^1^, Ruifang Zhang^2^, Yangbo Du^3^, Shuaihua Zhang^1^, Runze Gao^1^, Xuanqi Huang^1^, Qi Yang^3,^ *, Debin Kong^4, 5,^ * and Zhichang Xiao^1,^ *

^1^ Department of Chemistry, College of Science, Hebei Agricultural University, Baoding 071001, P. R. China

^2^ College of Land and Resources, Hebei Agricultural University, Baoding 071001, P. R. China

^3^ Key Laboratory of Civil Aviation Thermal Disaster Control and Emergency, Civil Aviation University of China, Tianjin 300300, P. R. China

^4^ Wuzhen Laboratory, Jiaxing 314500, P. R. China

^5^ College of New Energy, China University of Petroleum (East China), Qingdao 266580, P. R. China

* Corresponding authors. E-mail: [xiaozc@hebau.edu.cn](mailto:xiaozc@hebau.edu.cn) (Zhichang Xiao); [kongdb@upc.edu.cn](mailto:kongdb@upc.edu.cn) (Debin Kong); [q-yang@cauc.edu.cn](mailto:q-yang@cauc.edu.cn) (Qi Yang)

**S1 Materials synthesis**

**S1.1 Synthesis of Polypyrrole Nanotubes (PPyNT)**

In a typical procedure, 0.015 mol of anhydrous FeCl_3_ was added to a 300 mL aqueous solution containing 5 × 10^-3^ mol L^-1^ methyl orange (MO) under vigorous stirring. Subsequently, 0.015 mol of pyrrole monomer was introduced into the aforementioned solution, and the mixture was further stirred at room temperature for 24 h. After the reaction, the resulting black precipitate was washed with 1 mol L^-1^ HCl in a Soxhlet extractor for 24 h. The product was then dried under vacuum at 80 °C for 24 h to yield the black PPyNT powder.

**S1.2 Preparation of Heteroatom-doped Hierarchical Porous Carbon Materials (HHPC-x)**

Typically, 500 mg of the as-synthesized PPyNT was dispersed in a beaker containing 2.5 g FeCl_3_ and 2.5 g ZnCl_2_ (mass ratio of FeCl_3_ to ZnCl_2_ = 1:1), followed by the addition of 40 mL ethanol. The mixture was stirred until dissolution and ultrasonicated for 30 min. The solvent was then evaporated in an oil bath at 80 ⁰C, and the residue was transferred to an alumina boat and dried in a vacuum oven at 80 ⁰C for 24 h. The dried material was subjected to thermal treatment in a tube furnace under N_2_ atmosphere with a ramp rate of 5 ⁰C min^-1^: pre-carbonized at 400 ⁰C for 3 h, followed by pyrolysis at 800 ⁰C for 1 h. The resultant solid was collected by vacuum filtration with 1 mol L^-1^ HCl, washed in a Soxhlet extractor for 24 h, and dried under vacuum at 80 ⁰C for 24 h. The black product was ground to obtain HHPC-1. By varying the FeCl_3_/ZnCl_2_ mass ratios to 2:1 (HHPC-2) and 3:1 (HHPC-3) while maintaining the precursor-to-ZnCl_2_ mass ratio at 1:5 and other conditions unchanged, the corresponding HHPC-x samples were prepared via analogous thermal treatment of the polymerized precursors.

**S1.3 Preparation of HHPC without Molten Salt Treatment**

In a control experiment, 500.00 mg of PPyNT was dissolved in 40 mL anhydrous ethanol, and the subsequent operations followed the identical procedure as that for HHPC-x, yielding HHPC without molten salt activation.

**S2 Materials Characterizations**

The surface morphology and microstructure of the samples were examined using scanning electron microscopy (SEM, Hitachi 8010) and transmission electron microscopy (TEM, JEOL 4000EX). The crystallographic properties of the samples were analyzed by X-ray diffraction (XRD, Shimadzu XRD-6100) with a Cu Kα (λ = 1.5406 Å) radiation source. The surface chemical properties of the samples were investigated using X-ray photoelectron spectroscopy (XPS, America Thermo Fisher Scientific K-Alpha), Raman spectroscopy (514 nm, Horiba HR800), and Fourier transform infrared spectroscopy (FTIR, Bruker Equinox 55). The specific surface area and pore size distribution of the samples were determined by nitrogen adsorption-desorption isotherms at 77 K (Micromeritics ASAP2420). Static contact angle measurements were performed on a static contact angle measuring instrument (LAUDA Scientific, LSA100, Germany).

The details for the XPS spectra deconvolution are presented as follows: The high-resolution C 1s spectra (Fig. S2) are deconvoluted into five peaks: C=C/C-C (284.8 eV), C-N/C-O (285.8 eV), C-S (286.7 eV), C=O (287.7 eV), and O-C=O (288.9 eV) [S1]. The N 1s spectra (Fig. 2e) can be deconvoluted into pyridinic-N (398.3 eV), pyrrolic-N (399.5 eV), graphitic-N (400.8 eV), and oxidized-N (403.2 eV) [S2]. The O 1s spectra (Fig. 2f) are fitted to -C=O (531.3 eV), -C-O/C-O-C (532.3 eV), and O=C-O (533.6 eV) [S3]. The S 2p spectra (Fig. 2g) are fitted to C-S-C (S 2p_3/2_ at 162.4 eV and S 2p_1/2_ at 163.6 eV) and oxidized sulfur (C-SO_x_-C, peaks at 166.6 eV and 167.8 eV) [S4].

**S3 Electrochemical Measurements**

**S3.1 Preparation of Working Electrodes**

The working electrodes were fabricated by mixing HHPC-x, PVDF, and acetylene black in a mass ratio of 8.5:0.5:1.0, followed by grinding in N-methyl-2-pyrrolidone to form a homogeneous slurry. The slurry was then coated onto pre-treated titanium mesh and dried overnight at 100 ⁰C to obtain the working electrode.

**S3.2 Assembly of Zinc-Ion Capacitors**

Zinc-ion capacitors were assembled in CR2032 coin cells, with the prepared carbon electrode serving as the cathode, zinc foil as the anode, 1 mol L^-1^ ZnSO_4_ aqueous solution as the electrolyte, and glass microfiber as the separator.

**S3.3 Electrochemical Performance Evaluation**

Cyclic voltammetry (CV), galvanostatic charge-discharge (GCD), and electrochemical impedance spectroscopy (EIS) measurements were conducted using a CHI 760E electrochemical workstation (CH Instruments, Inc., China). The voltage window was set from 0.2 to 1.8 V (vs. Zn^2+^/Zn), with EIS performed over a frequency range of 0.01-100,000 Hz and an amplitude of 10 mV. Long-term GCD cycling tests were carried out on a NEWARE CT4008 battery testing system (Neware Technology Ltd., Shenzhen, China).

The specific capacity (C, mAh g^-1^) of the zinc-ion capacitors was calculated according to Equation (S1):

$C=2I ʃ Vdt/3.6Vm$ (S1)

where *I* (A) is the discharge current, *ʃ 𝑉𝑑𝑡* (Vs) is the integrated area under the discharge curve, *V* (V) is the discharge voltage after iR drop, and *m* (g) is the mass of the active material in the cathode.

The energy density (*E*, Wh kg^-1^) and power density (*P*, W kg^-1^) of the ZICs were determined using Equations (S2) and (S3):

$E=I ʃ Vdt/3.6m$ (S2)

$P=3600E/\Delta t$ (S3)

where *I* (A), ∆t (s), *m* (g), and *V* (V) represent the discharge current, discharge time, mass of the active material on the cathode, and discharge voltage, respectively.

To investigate the charge storage kinetics, the relationship between peak current (*i*) and scan rate (*v*) was analyzed using Equation (S4):

$i=av^{b}$ (S4)

where *a* and *b* are adjustable constants, *i* is the current, and *v* is the scan rate.

The contributions from capacitive (fast) and diffusion-controlled (slow) processes were quantified using the Dunn’s method, based on the relationship between current (*i*) and scan rate (*v*) as described by Equations (S5) and (S6):

$i=k_{1}v+k_{2}v^{1/2}$ (S5)

$\frac{i}{v^{1/2}}=k_{1}v^{1/2}+k_{2}$ (S6)

where *k_1_* and *k_2_* are constants; the term *k_1_v* corresponds to the capacitive contribution, and *k_2_v* represents the diffusion-controlled contribution.

The ion diffusion coefficient was derived from EIS data using Equation (S7):

$Z^{'}={\sigma\omega}^{-0.5}+Re+Rct$ (S7)

where Z’ is the real part of the impedance, ω is the angular frequency (rad s^-1^), σ is the Warburg coefficient (Ω s^-1/2^), R_e_ is the equivalent series resistance (Ω), and R_ct_ is the charge transfer resistance (Ω).

The self-charging performance was evaluated in terms of the self-charging efficiency (η) and the average self-charging rate (ν), as follows:

$\eta=\frac{C_{self-charging}}{C_{galvanostatic}}\times100 \%$ (S8)

$\nu=\frac{C_{cyclic self-charging}}{T_{total}}$ (S9)

where C_self-charging_, C_galvanostatic_, and C_cyclic self-charging_ represent self-charging discharge capacity, galvanostatic discharge capacity, and total discharge capacity after cyclic self-charging. *T*_total_ represents the total cyclic self-charging time.

**S4 Computational details**

**S4.1 Finite element simulation**

In this study, three geometric models with hierarchical pore structures were constructed for the carbon-based nanosheets and Finite Element Analysis (FEA) was conducted utilizing the COMSOL Multiphysics 6.2 software. The computational domain comprises the upper and lower surfaces of the nanosheets and the internal porous channels. The number of hierarchical pore channels were determined by referencing the proportions of S_0.4-0.86nm_, S_0.86-2nm_ and S_>2nm_. The initial molar concentration of [Zn(H_2_O)_6_]^2+^ at the upper surface was specified as 2 mol/L. To elucidate the underlying mechanism of micro-geometric constraints on the diffusion of [Zn(H_2_O)_6_]^2+^, such as the concentration gradient and flux distribution, the simulation was implemented using the “Transport of Diluted Species” interface. The mass transfer behavior of [Zn(H_2_O)_6_]^2+^ ions within the electrolyte-filled pores is governed by Fick’s second law:

$$\frac{\text{∂}\text{c}_{\text{i}}}{\text{∂}\text{t}}\text{+∇∙}\text{J}_{\text{i}}\text{+u∙∇}\text{c}_{\text{i}}\text{=}\text{R}_{\text{i}}$$

$$\text{J}_{\text{i}}\text{=-}\text{D}_{\text{i}}\text{∇}\text{c}_{\text{i}}$$

Where $\text{c}_{\text{i}}$ (mol/m^3^) represents the instantaneous molar concentration of [Zn(H_2_O)_6_]^2+^, $\text{J}_{\text{i}}$ mol/(m^2^·s) is the diffusion flux,*u* (m/s) is the velocity vector, $\text{R}_{\text{i}}$ mol/(m^3^·s) is the source term defined as the net production or net consumption of a substance per unit volume per unit time, and $\text{D}_{\text{i}}$ (m^2^/s) is the diffusion coefficient of [Zn(H_2_O)_6_]^2+^ in the electrolyte inside the pore channels.

**S4.2 DFT method**

All density functional theory (DFT) computations were performed using the Vienna Ab initio Simulation Package (VASP) [S5, S6]. The Perdew-Burke-Ernzerhof (PBE) functional within the generalized gradient approximation (GGA) was employed to treat exchange-correlation effects [S7]. Electron-ion interactions were modeled with the projector augmented-wave (PAW) method [S8]. A plane-wave energy cutoff of 400 eV was applied, and the Brillouin zone was sampled using a 3 × 3 × 1 K-point. Geometry optimizations were considered converged when the total energy change between successive cycles fell below 10^-5^ eV and the residual forces on all atoms were less than 0.02 eV Å^-1^. To properly account for dispersion interactions, the DFT-D3 correction scheme was incorporated [S9]. Structural models and simulation results were visualized using the VESTA software [S10].

The energy associated with the complete desolvation of a hydrated zinc ion, defined as the desolvation energy (*E*_d_), was computed according to:

*E*_d_ = *E*(Zn^2+^) + 6*E*_H2O_ - *E*{[Zn(H_2_O)_6_]^2+^}

To evaluate the thermodynamic favorability under realistic conditions, the Gibbs free energy change for desolvation (*ΔG*_d_) was further determined using:

*ΔG*_d_ = *G*(Zn^2+^) + 6*G*_H2O_ - *G*{[Zn(H_2_O)_6_]^2+^}

The Gibbs free energy (G) for each species was derived from its electronic energy with vibrational corrections:

*G* = *E* + ZPE - *TS*

where ZPE denotes the zero-point energy, S is the entropy, and T represents the temperature (set to 298.15 K). The ZPE and S were obtained from frequency calculations performed on the optimized structures.

**S5 Supplementary Figures and Tables**


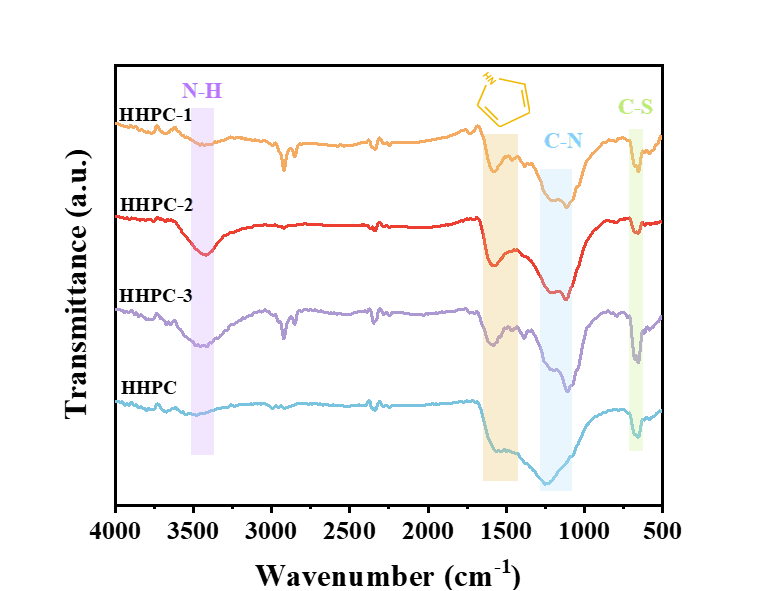


**Fig. S1** FTIR Spectra of HHPC and HHPC-x


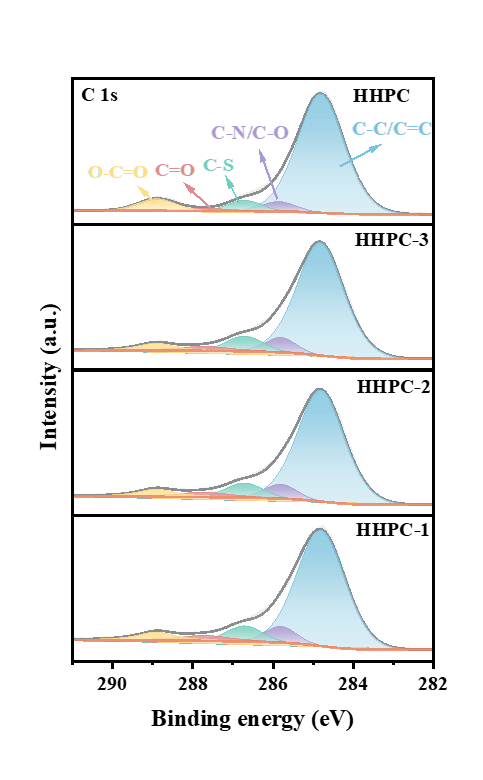


**Fig. S2** C 1s XPS spectra analysis of HHPC and HHPC-x samples


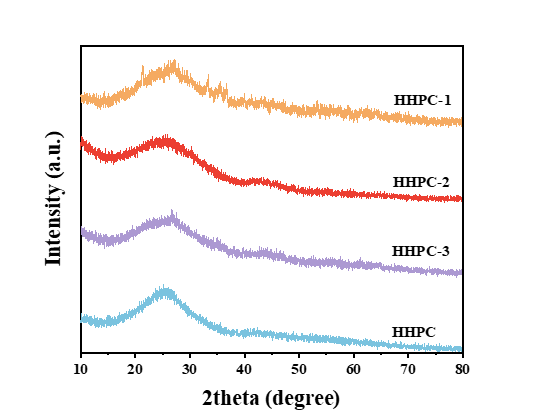


**Fig. S3** XRD patterns of HHPC and HHPC-x samples


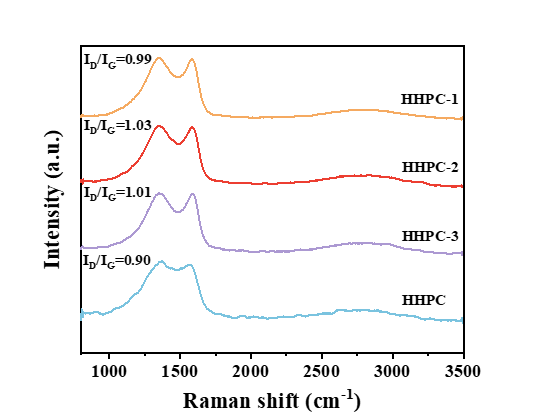


**Fig. S4** Raman spectra of HHPC and HHPC-x samples


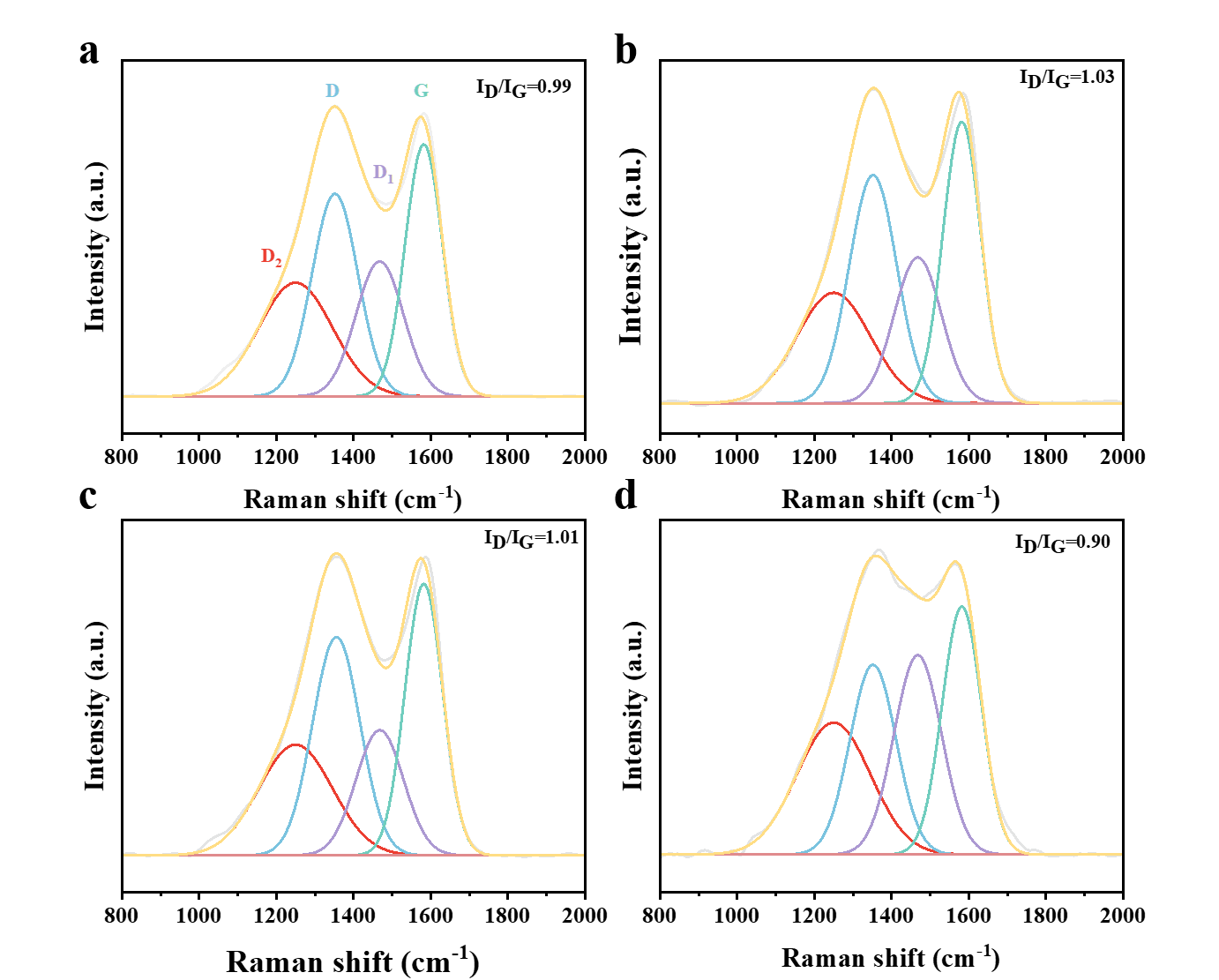


**Fig. S5** Fitted Raman spectra of (**a**) HHPC-1, (**b**) HHPC-2, (**c**) HHPC-3, and (**d**) HHPC


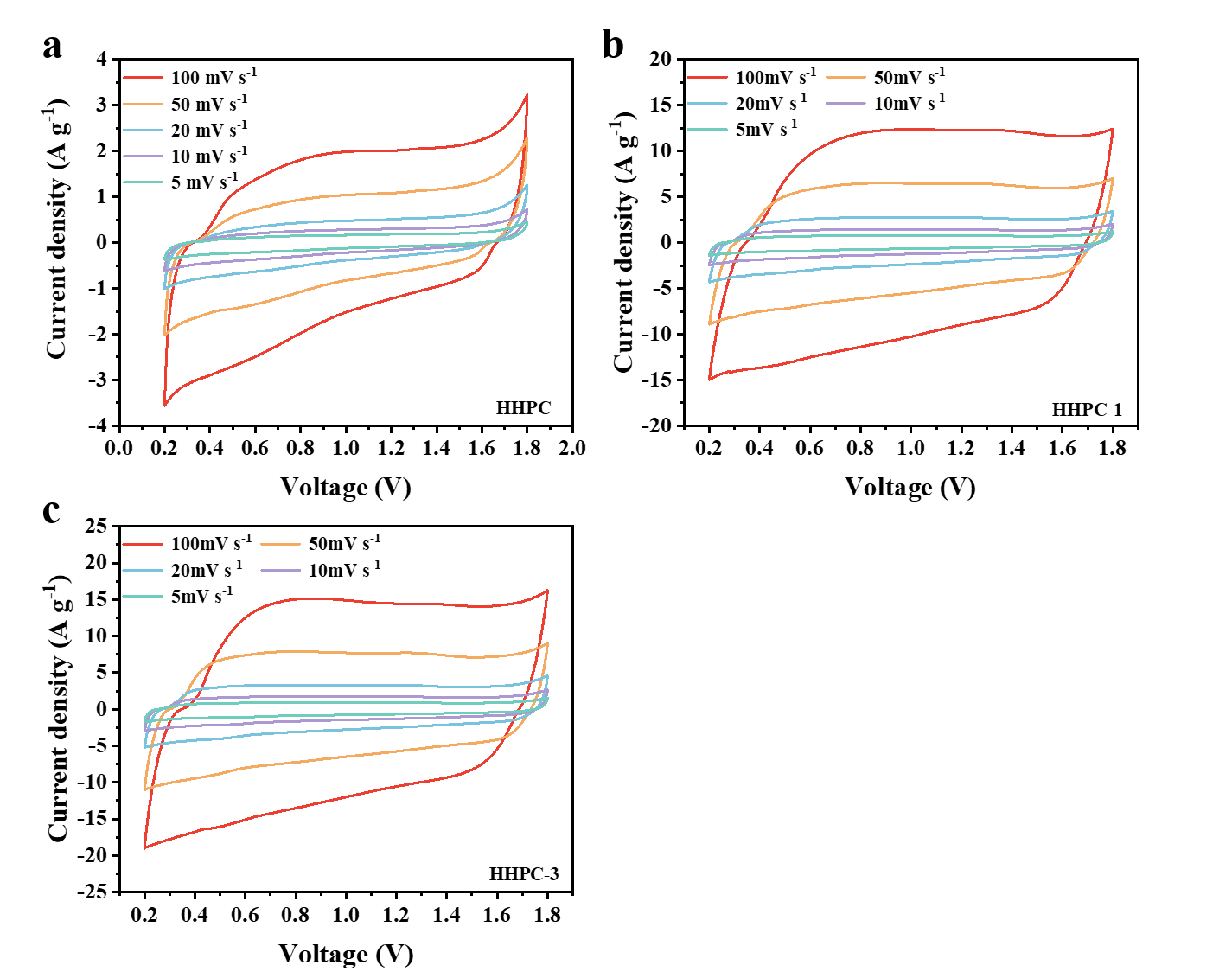


**Fig. S6** CV curves of (**a**) HHPC, (**b**) HHPC-1, and (**c**) HHPC-3 with a scan rate range of 5-100 mV s^-1^


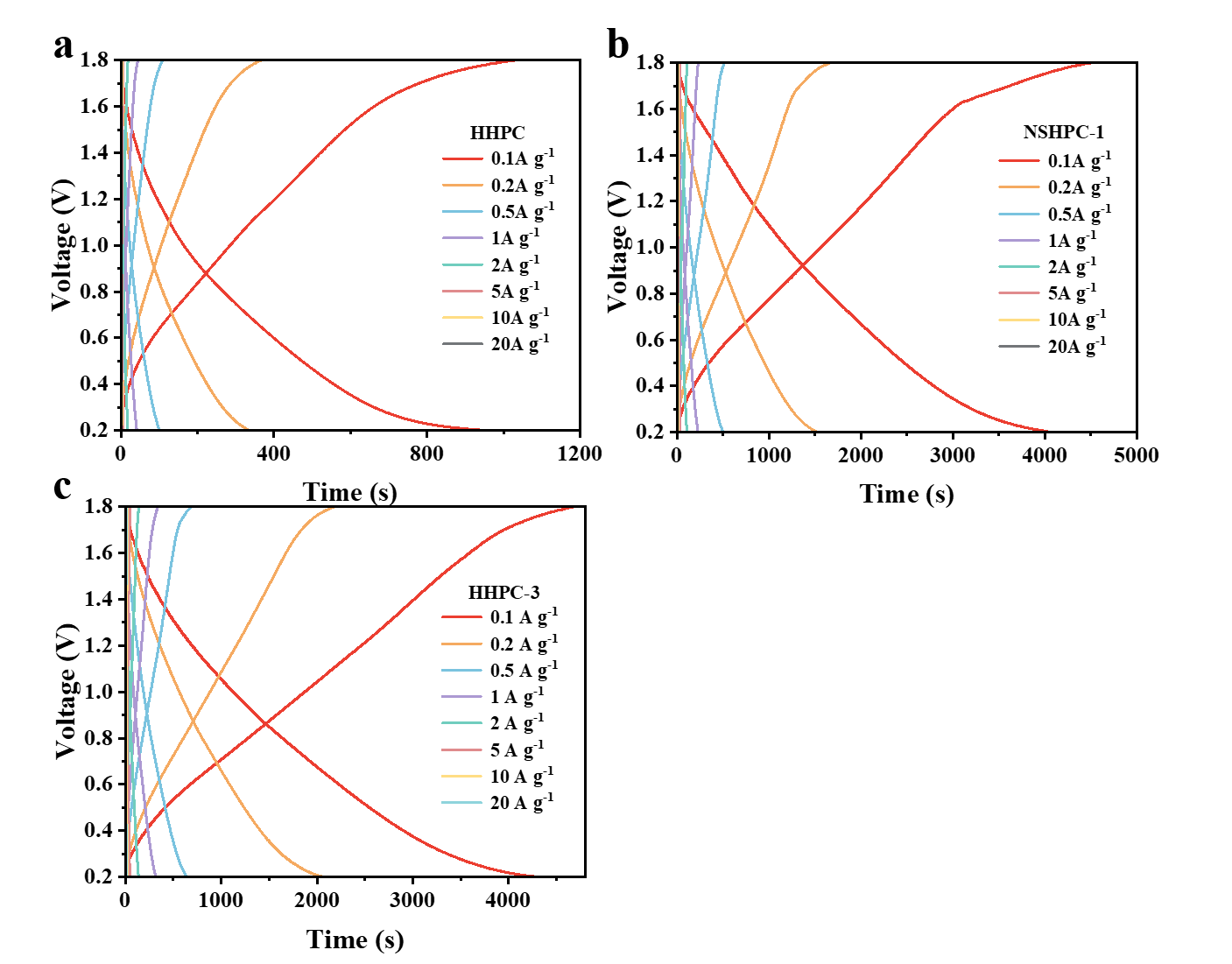


**Fig. S7** GCD curves of (**a**) HHPC, (**b**) HHPC-1 and (**c**) HHPC-3 at the current densities from 0.1 to 20 A g^-1^


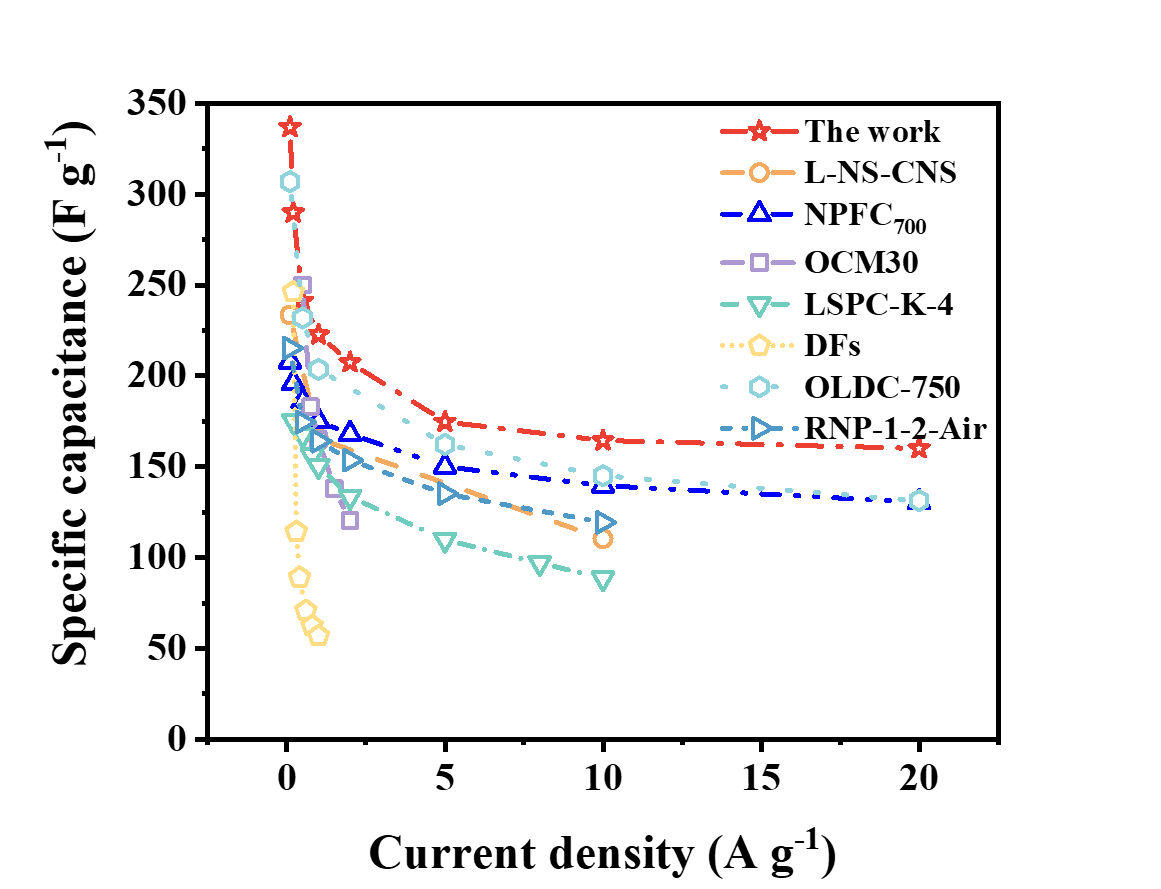


**Fig. S8** Capacitance comparison between HHPC-2 cathode and state-of-art reported carbon electrodes


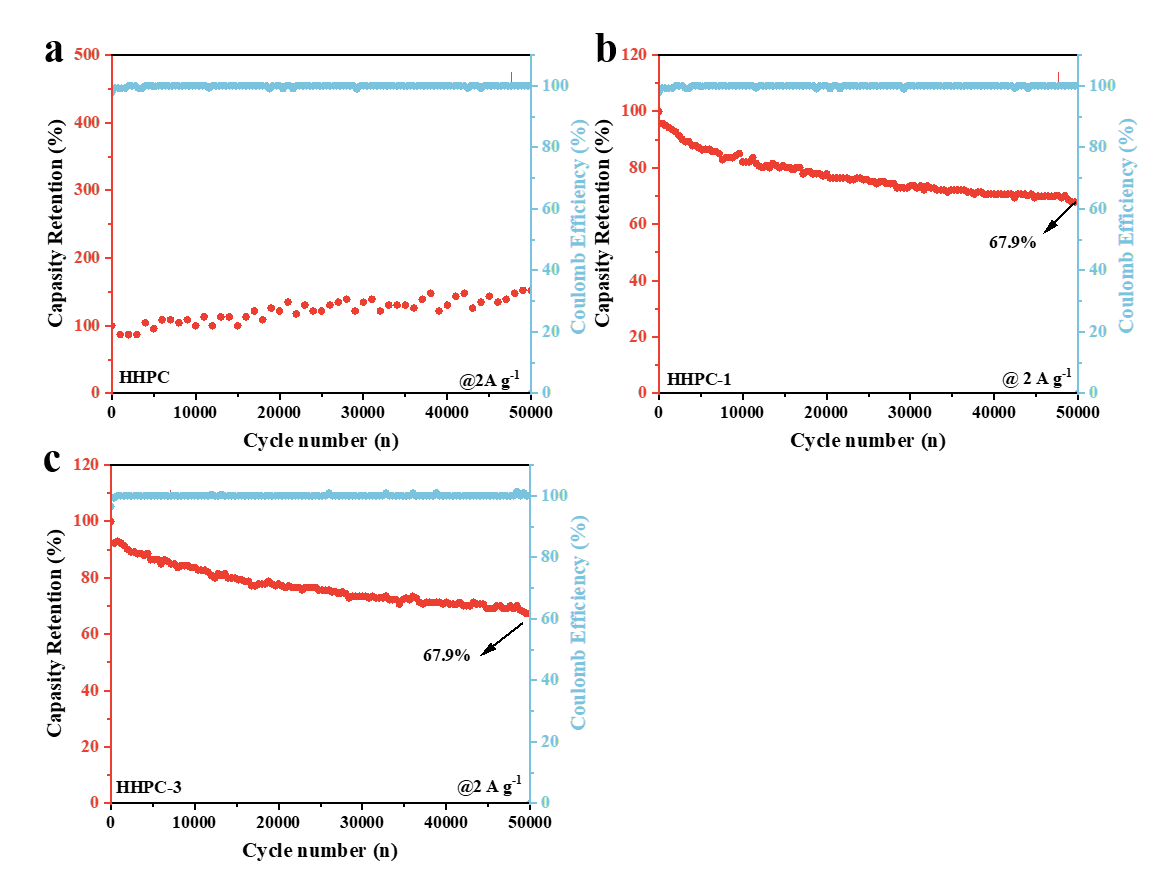


**Fig. S9** Cycling stability of (**a**) HHPC, (**b**) HHPC-1, (**c**) HHPC-3 at 2 A g^-1^


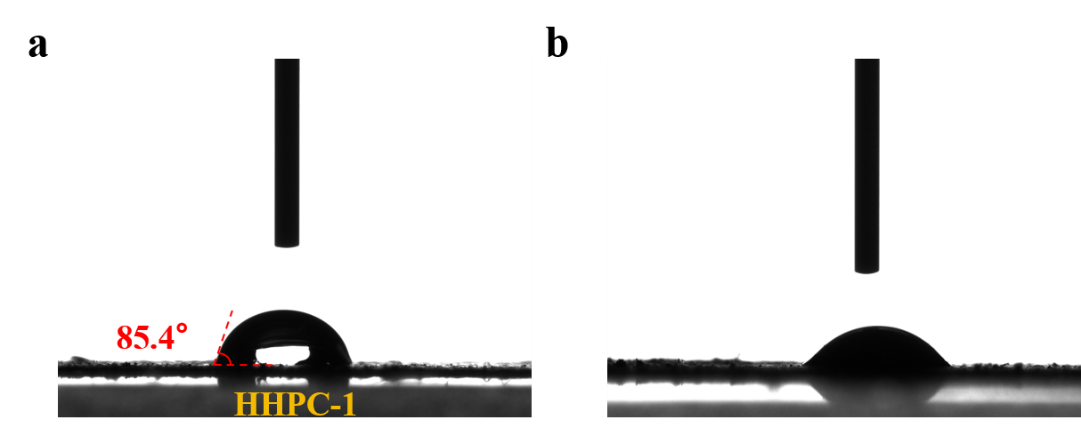


**Fig. S10** The contact angle tests of (**a**) HHPC-1 and (**b**) HHPC-3


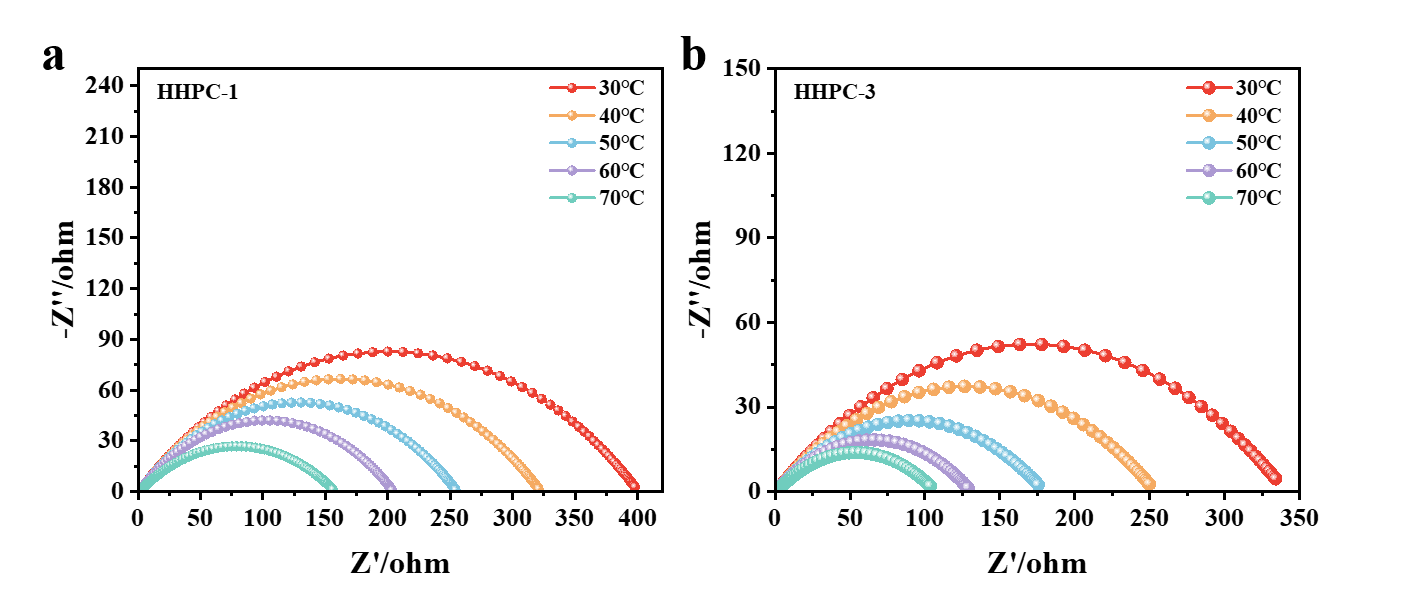


**Fig. S11** EIS spectra at different temperatures of the Zn//Zn symmetric cells with a (**a**) HHPC-1 and (**b**) HHPC-3 interfacial layer


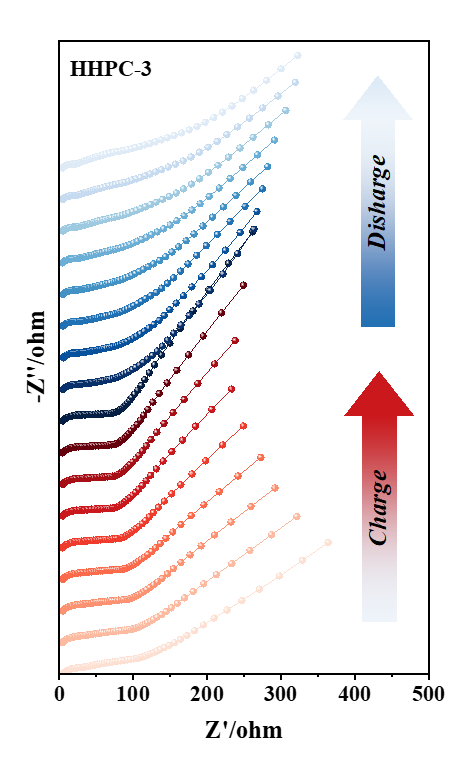


**Fig. S12** Nyquist plots of HHPC-3 from the in-situ EIS measurements


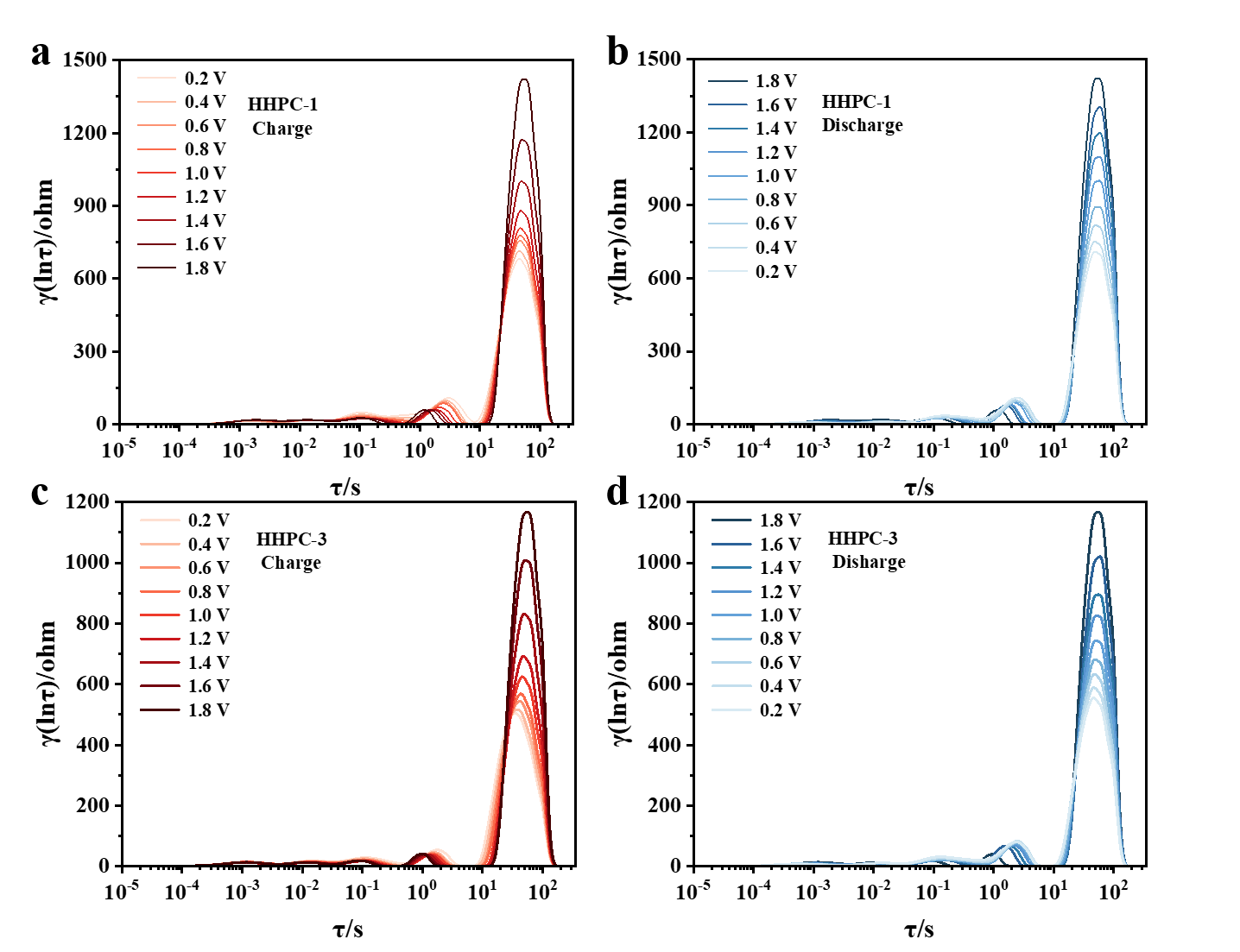


**Fig. S13** DRT calculated from EIS measurements of (**a)-(b**) HHPC-1 and (**c)-(d**) HHPC-3 at different potentials


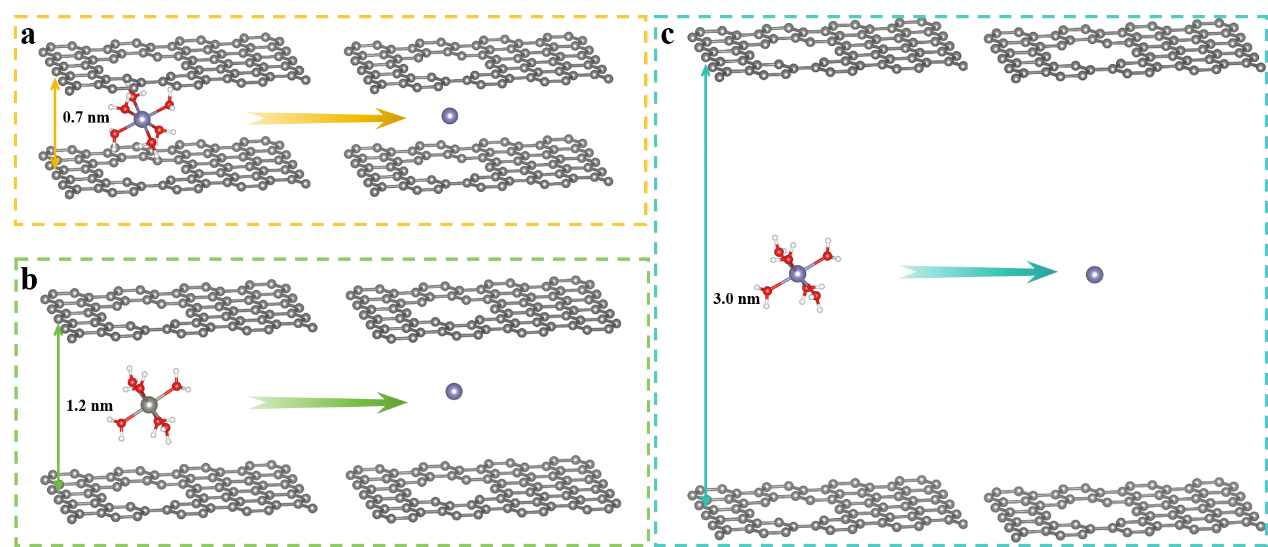


**Fig. S14** The model structures for the desolvation of [Zn(H_2_O)_6_]^2+^ ions based on double-layer graphene with layer distances of (**a**) 0.7 nm, (**b**) 1.2 nm and (**c**) 3.0 nm


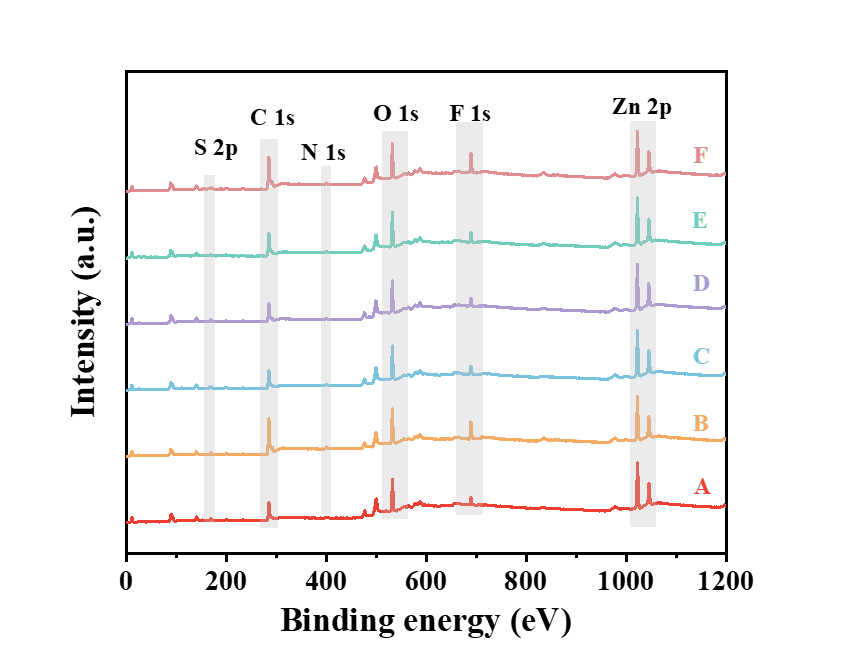


**Fig. S15** Ex-situ XPS survey spectra of HHPC-2 cathode at different stages


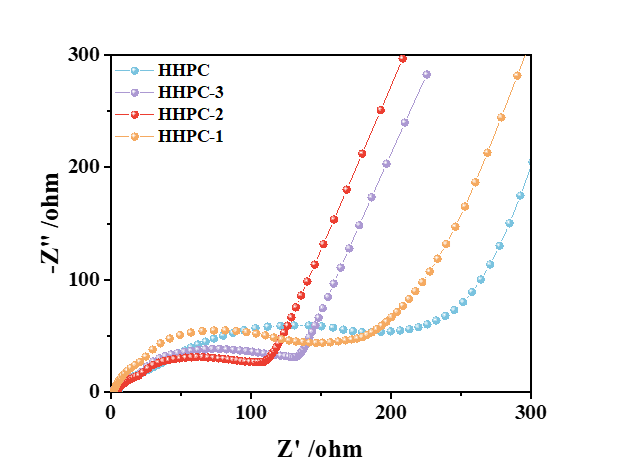


**Fig. S16** Nyquist plots of HHPC and HHPC-x samples


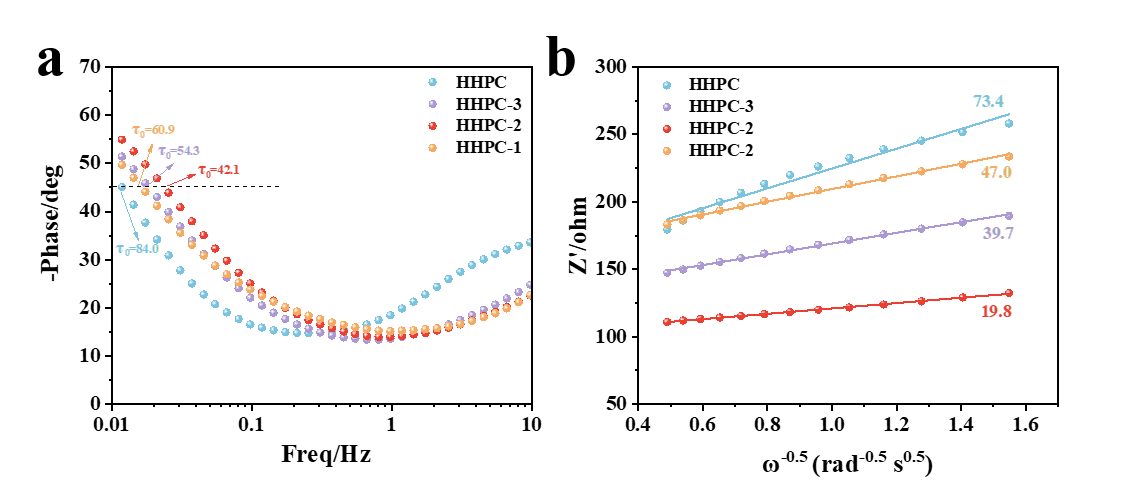


**Fig. S17** (**a**) Frequency and phase angle diagram; (**b**) Linear plots of real resistances (Z’) against angular frequencies (ω^−0.5^) in the low-frequency region


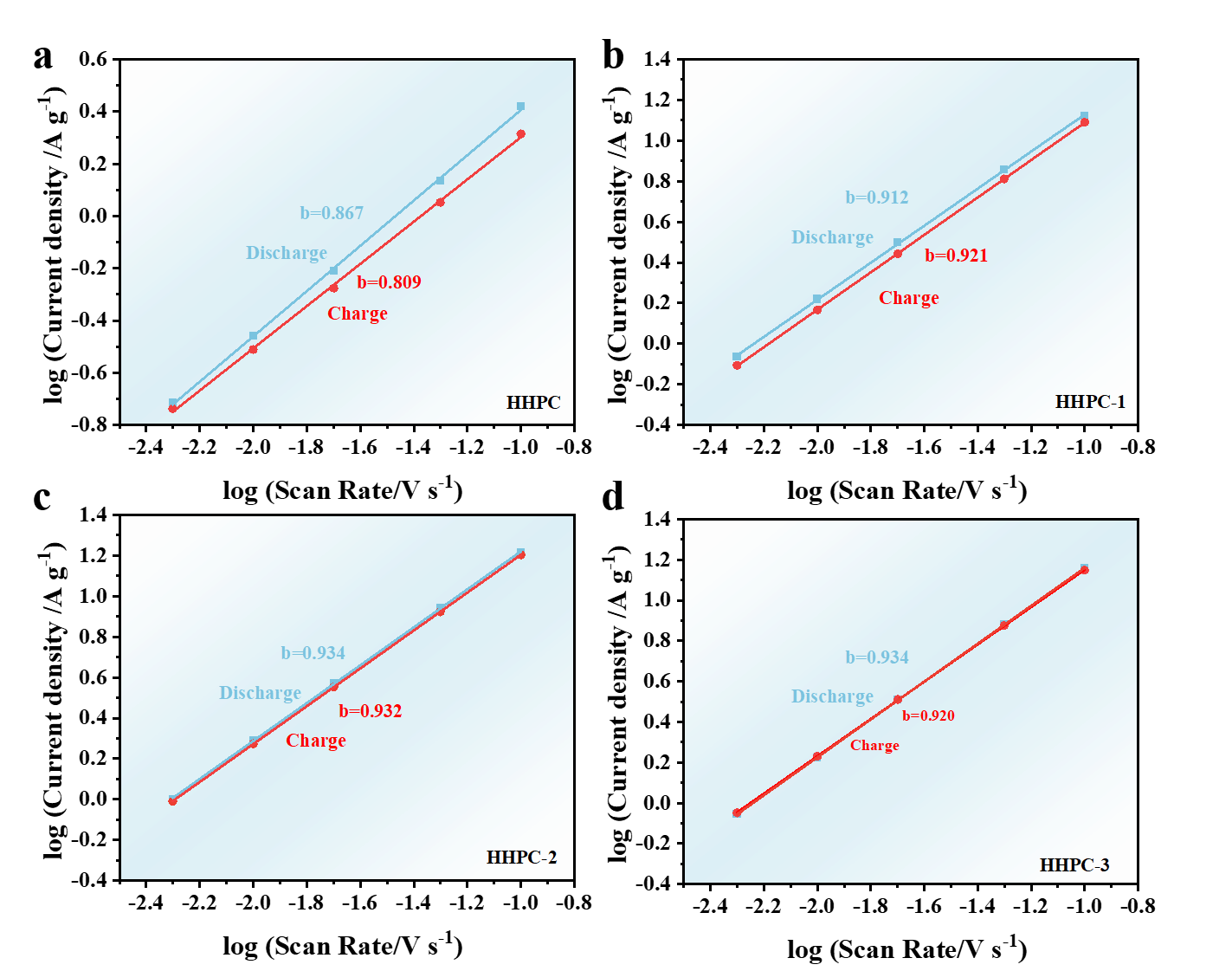


**Fig. S18** The fitting plots between log (i) and log (v) for (**a**) HHPC; (**b**) HHPC-1; (**c**) HHPC-2 and (**d**) HHPC-3


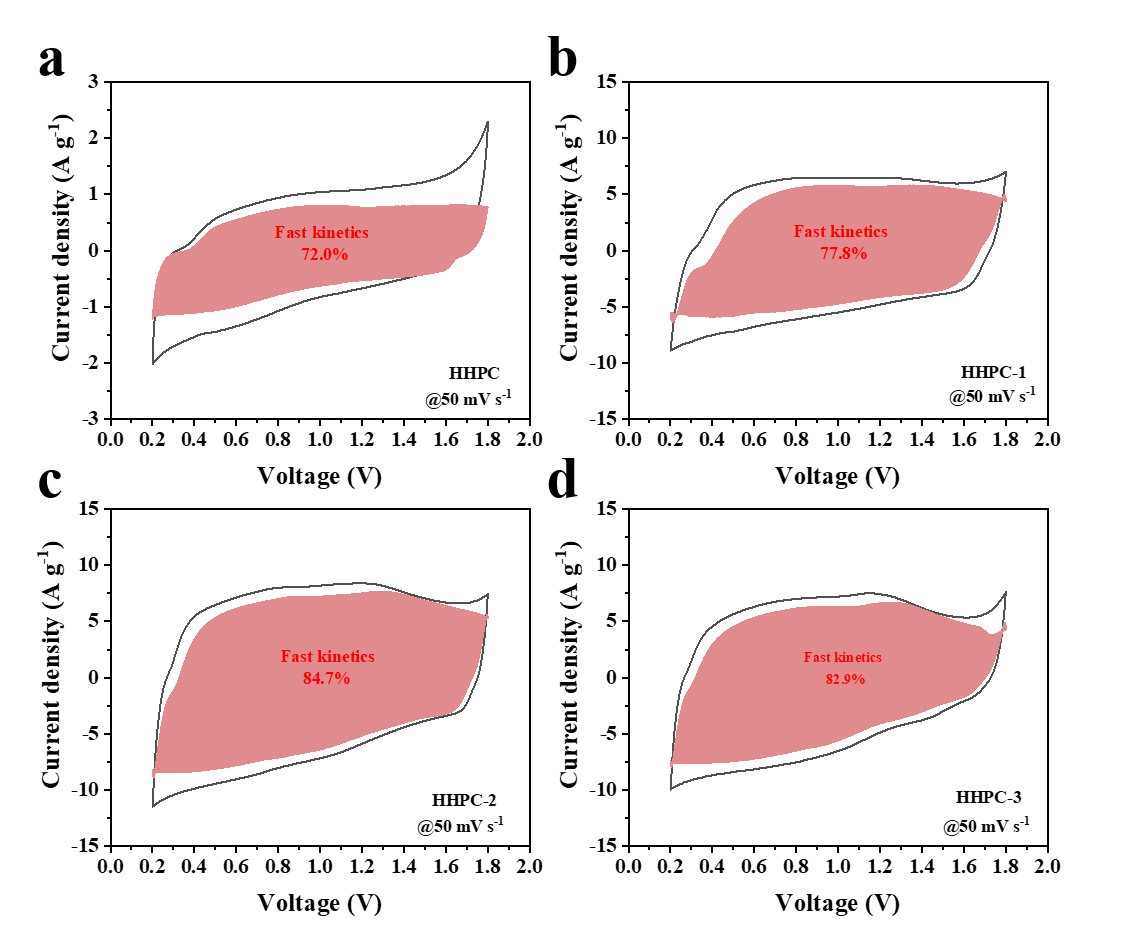


**Fig. S19** The calculated capacitive contributions at a scanning rate of 50 mV s^-1^ for (**a**) HHPC; (**b**) HHPC-1; (**c**) HHPC-2 and (**d**) HHPC-3


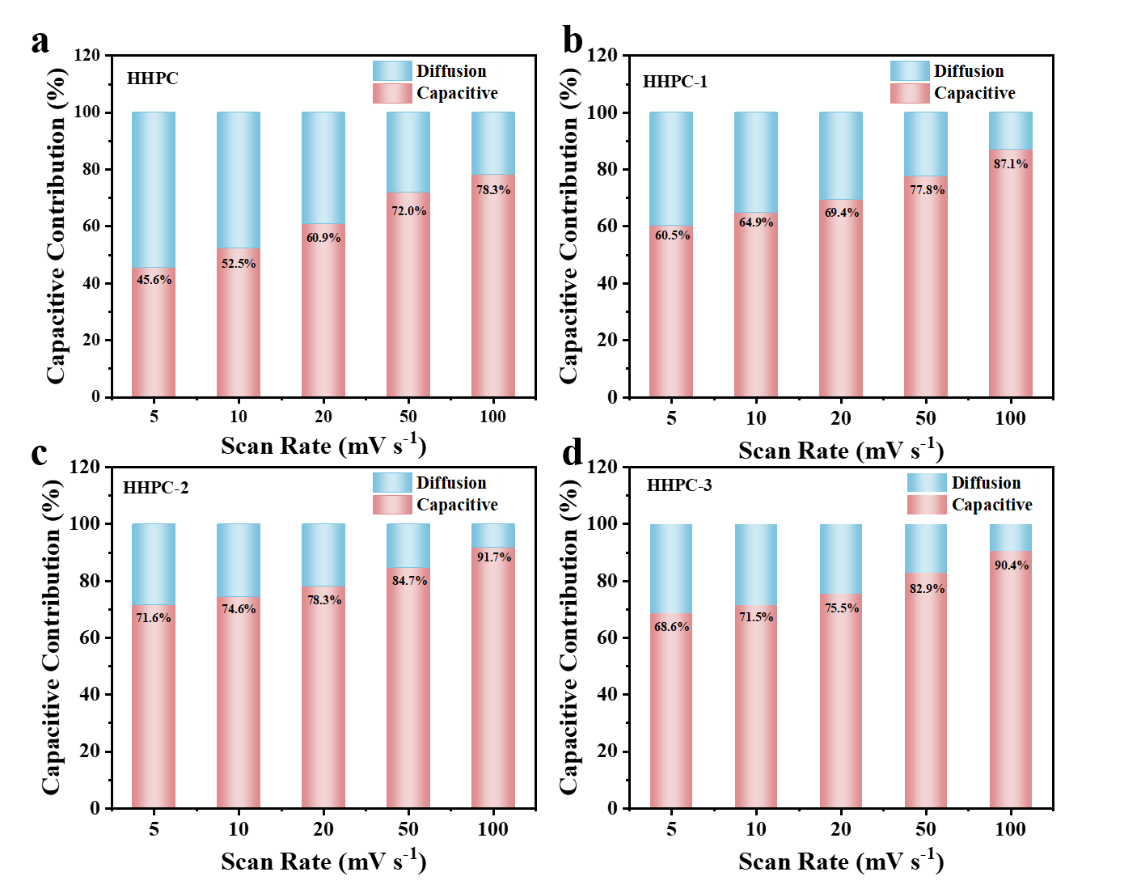


**Fig. S20** The calculated capacitive contribution of (**a**) HHPC; (**b**) HHPC-1; (**c**) HHPC-2 and (**d**) HHPC-3 electrodes at various scan rates from 5 to 100 mV s^-1^


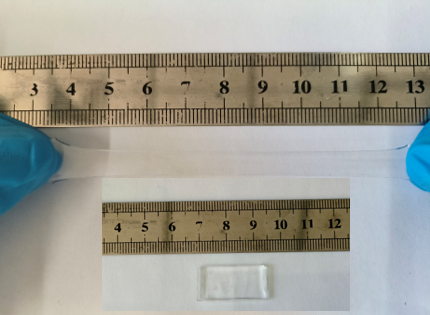


**Fig. S21** Photograph of the Zn(CF_3_SO_3_)_2_/PVA gel electrolyte


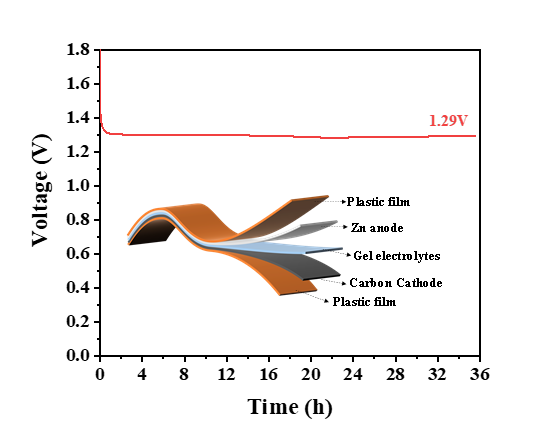


**Fig. S22** Self-discharge performance (inset is the illustration for the assembly of FZIC) of HHPC-2-based FZIC


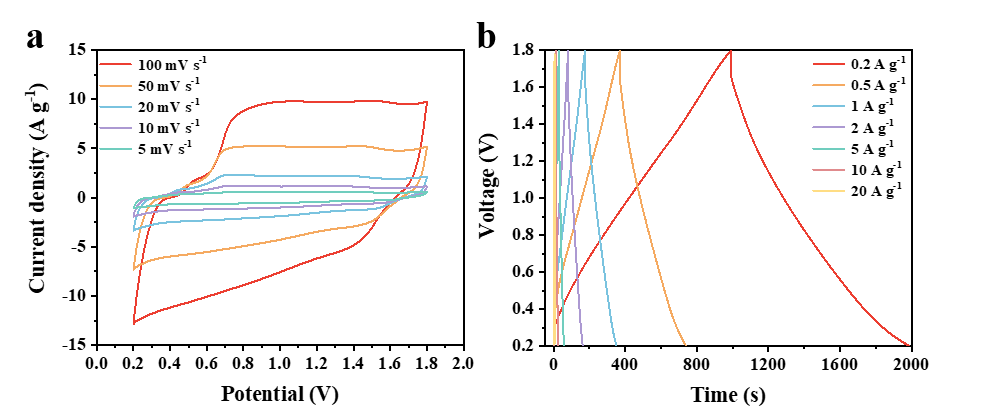


**Fig. S23** (**a**) CV curves and (**b**) GCD curves of HHPC-2-based FZIC


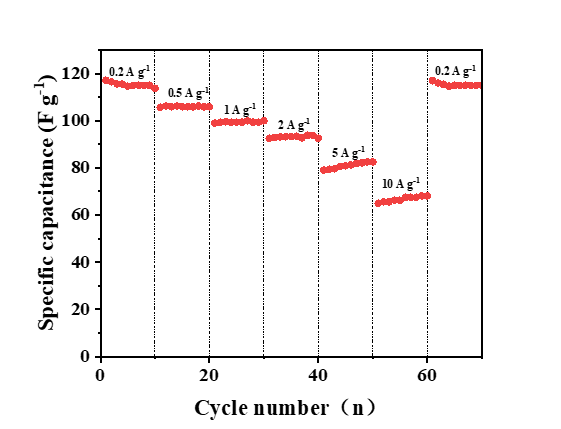


**Fig. S24** Rate capability of HHPC-2-based FZIC


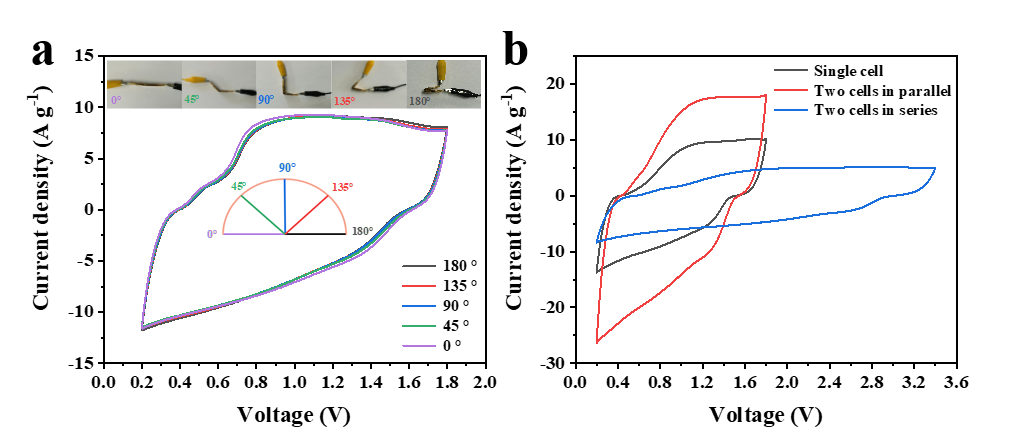


**Fig. S25** (**a**) CV curves at different bending angles and (**b**) CV curves of the devices connected in parallel and series for HHPC-2-based FZIC


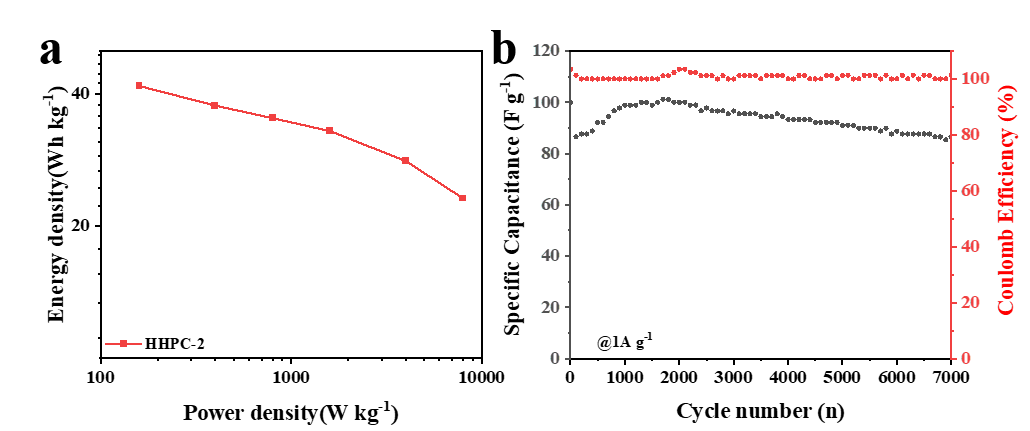


**Fig. S26** (**a**) Ragone plot and (**b**) cycling stability at 1 A g^-1^ for HHPC-2-based FZIC


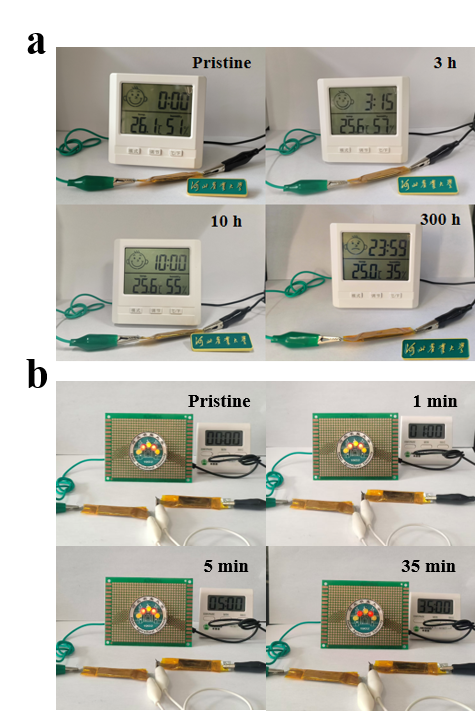


**Fig. S27** (**a**) A single FZIC powering a timer; (**b**) Two FZICs connected in series powering five LED lights


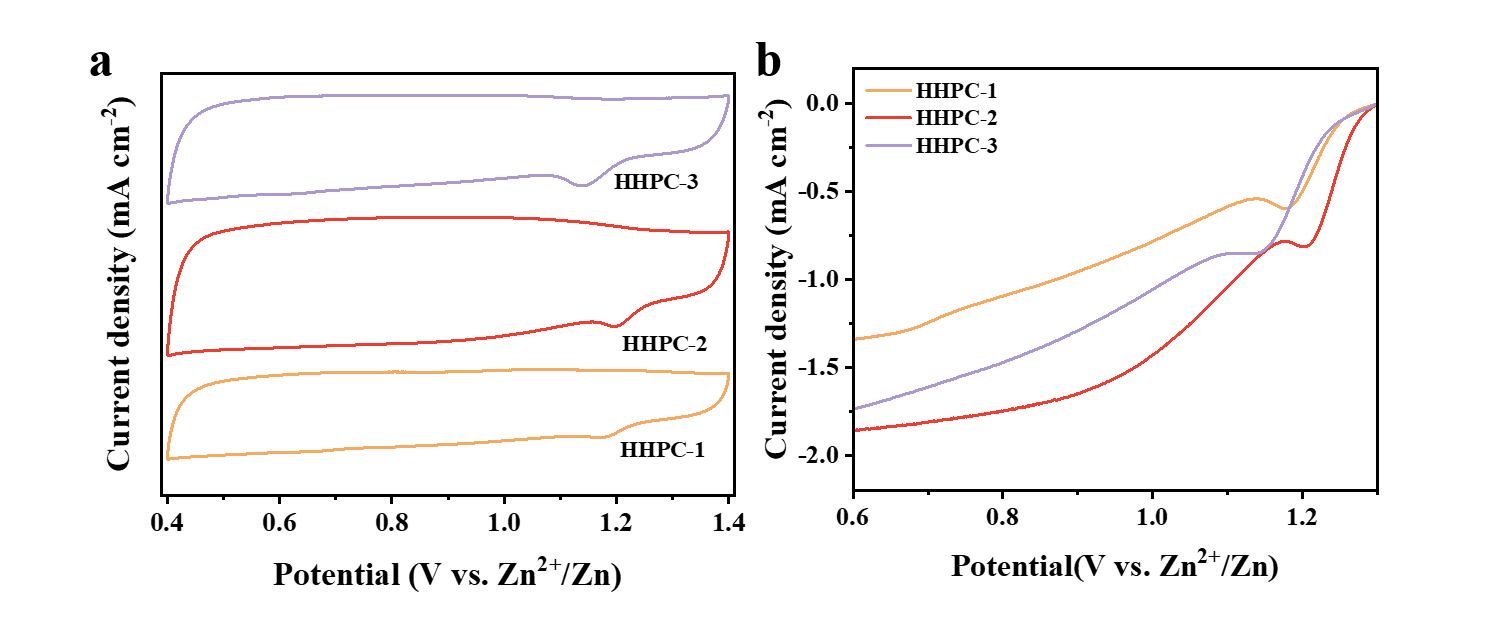


**Fig. S28** (**a**) CV curves tested in O_2_-saturated 1M ZnSO_4_; (**b**) ORR polarization curves of HHPC-1, HHPC-2 and HHPC-3


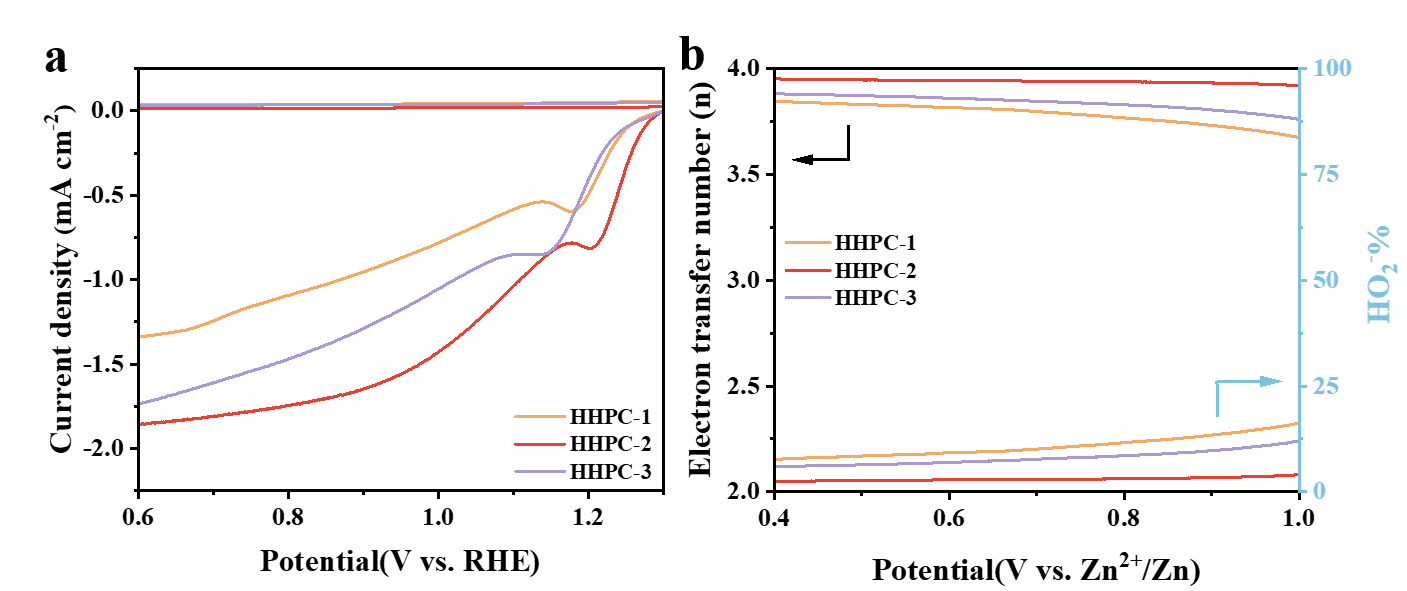


**Fig. S29** (**a**) ORR polarization curves of HHPC-1, HHPC-2 and HHPC-3 measured on RRDE in O_2_-saturated 1M ZnSO_4_; (**b**) The electron transfer number and HO_2_^-^ yield calculated from (a)

**Table S1** SSA parameters of HHPC and HHPC-x

| **Sample** | **S_BET_**  **(m^2^ g^-1^)** | | **V_Total_**  **(cm^3^ g^-1^)** | **S_micro_**  **(m^2^ g^-1^)** | **S_0.4-0.86nm_**  **(m^2^ g^-1^)** | **S_0.86-2nm_**  **(m^2^ g^-1^)** | **S_meso_**  **(m^2^ g^-1^)** | **S_0.4-0.86nm_**  **/S_BET_** | **S_0.86-2nm_**  **/S_BET_** | **S_>2nm_**  **/S_BET_** |
| --- | --- | --- | --- | --- | --- | --- | --- | --- | --- | --- |
| HHPC-3 | | 2325.3 | 1.71 | 1554.8 | 261.7 | 423.4 | 770.5 | 21.0% | 34.0% | 45.0% |
| HHPC-2 | | 2523.0 | 1.87 | 1878.3 | 458.7 | 459.3 | 644.5 | 30.6% | 30.7% | 38.7% |
| HHPC-1 | | 1810.0 | 1.36 | 1224.4 | 598.1 | 428.0 | 585.6 | 46.4% | 33.2% | 20.4% |
| HHPC | | 43.6 | 0.13 | 12.5 | 0 | 0 | 31.1 | 0 | 0 | 100% |

**Table S2** Oxygen configuration analysis of HHPC and HHPC-x

| **Binding**  **Energy(eV)** | **Oxygen**  **Bonding** | **Relative Concentration** | | | | |
| --- | --- | --- | --- | --- | --- | --- |
|  |  | **HHPC-3** | | **HHPC-2** | **HHPC-1** | **HHPC** |
| 531.3 | -C=O | 0.34 | 0.32 | | 0.31 | 0 |
| 532.3 | -C-OH/C-O-C | 0.42 | 0.46 | | 0.45 | 0.67 |
| 533.6 | O=C-O | 0.24 | 0.23 | | 0.21 | 0.33 |

**Table S3** Nitrogen configuration analysis of HHPC and HHPC-x

| **Binding**  **Energy(eV)** | **Nitrogen**  **Bonding** | **Relative Concentration** | | | |
| --- | --- | --- | --- | --- | --- |
|  |  | **HHPC-3** | **HHPC-2** | **HHPC-1** | **HHPC** |
| 398.3 | Pyridinic N | 0.26 | 0.26 | 0.27 | 0.41 |
| 399.5 | Pyrrolic N | 0.21 | 0.19 | 0.17 | 0.15 |
| 400.8 | Graphitic N | 0.48 | 0.50 | 0.52 | 0.44 |
| 403.2 | N-oxides | 0.05 | 0.04 | 0.05 | 0.00 |

**Table S4** Comparison of the capacitance of HHPC and HHPC-x at different current densities

| **Sample** | **0.1 A g^-1^**  **(F g^-1^)** | **0.2 A g^-1^**  **(F g^-1^)** | **0.5 A g^-1^**  **(F g^-1^)** | **1.0 A g^-1^**  **(F g^-1^)** | **2.0 A g^-1^**  **(F g^-1^)** | **5.0 A g^-1^**  **(F g^-1^)** | **10.0 A g^-1^**  **(F g^-1^)** | **20.0 A g^-1^**  **(F g^-1^)** | **Capacity**  **retention** |
| --- | --- | --- | --- | --- | --- | --- | --- | --- | --- |
| HHPC-3 | 270.0 | 258.2 | 200.9 | 192.1 | 171.5 | 145.0 | 129.4 | 112.5 | 41.7% |
| HHPC-2 | 336.9 | 289.8 | 241.3 | 222.6 | 207.4 | 174.7 | 164.4 | 160.0 | 47.5% |
| HHPC-1 | 255.0 | 191.0 | 156.9 | 142.6 | 133.1 | 106.9 | 98.1 | 87.5 | 34.3% |
| HHPC | 59.9 | 42.0 | 31.4 | 26.0 | 22.6 | 18.4 | 16.2 | 15.0 | 25.1% |

**Table S5** Comparison of the performance of HHPC-2-based ZIC with recently reported carbon-based ZICs

| **Materials** | **Electrolyte** | **Voltage**  **(V)** | **Current density**  **(A g^-1^)** | **Capacity** | **Power density**  **(W kg^-1^)** | **Energy density**  **(Wh kg^-1^)** | **Refs.** |
| --- | --- | --- | --- | --- | --- | --- | --- |
| L-NS-CNS | 1 M ZnSO_4_ | 0.2-1.8V | 0.1 | 233.4 F g^-1^ | 94 | 91 | [S11] |
| NPFC700 | 1 M Zn(CF_3_SO_3_)_2_ | 0.2-1.8 V | 0.1 | 207.9 F g^-1^ | 15400 | 80 | [S12] |
| OCM30 | 1 M ZnSO_4_ | 0.2-1.8 V | 2 | 120.4 F g^-1^ | 1699.8 | 51.9 | [S13] |
| LSPC-K-4 | 1 M ZnSO_4_ | 0.2-1.8 V | 0.2 | 175.1 F g^-1^ | 160.0 | 62.3 | [S14] |
| RFK5 | 2 M ZnSO_4_ | 0.2-1.8 V | 0.5 | 101 mAh g^-1^ | 391 | 79 | [S15] |
| DFs | 1 M ZnSO_4_ | 0.2-1.8 V | 0.2 | 246.1 F g^-1^ | 709.0 | 70.7 | [S16] |
| OLDC-750 | 2 M ZnSO_4_ | 0.2-1.8 V | 0.1 | 306.8 F g^-1^ | 20000 | 58.3 | [S17] |
| RNP-1-2-Air | 2 M ZnSO_4_ | 0.2-1.8 V | 0.1 | 215.2 F g^-1^ | 4000 | 54.4 | [S18] |
| N-OPCNF | 1 M ZnSO_4_ | 0.2-1.8 V | 0.1 | 136 mAh g^-1^ | 72.27 | 98.28 | [S19] |
| HHPC-2 | 1 M ZnSO_4_ | 0.2-1.8 V | 0.1 | 336.9 F g^-1^ | 80.0 | 120.0 | **This work** |

**Supplementary References**

1. X. Mou, Q. Yang, B. Zhao, X. Xin, J. Zhang et al., Graphitic carbocatalysts with pentagon defects synthesized from polyperylene for electrocatalytic oxygen reduction and flexible zinc-air batteries. ACS Appl. Nano Mater. **7**(12), 14526–14539 (2024). <https://doi.org/10.1021/acsanm.4c01965>
2. Z. Xiao, X. Mou, X. Meng, Q. Yang, Y. Ma et al., Electrifying Schiff-based networks as model catalysts towards deeply understanding the crucial role of sp2-carbon in nitrogen-doped carbocatalyst for oxygen reduction reaction. Appl. Surf. Sci. **599**, 153961 (2022). <https://doi.org/10.1016/j.apsusc.2022.153961>
3. [3] J. Zhang, R. Zhang, S. Zhang, R. Gao, Q. Yang et al., Integrating optimized pore structure and enriched carbonyl groups into functional carbon nanomaterials for high-performance zinc-ion capacitors. Chin. Chemical Lett., 111867 (2025). <https://doi.org/10.1016/j.cclet.2025.111867>
4. Q. Wang, S. Zhang, J. Zhang, R. Gao, Y. Sun et al., Deciphering structure–activity relationships in sulfur-doped carbon nanomaterials through tailored synthesis pathways for efficient electrocatalytic oxygen reduction. ACS Appl. Nano Mater. **8**(33), 16465–16475 (2025). <https://doi.org/10.1021/acsanm.5c02781>
5. G. Kresse, J. Hafner, *Ab initio*molecular dynamics for liquid metals. Phys. Rev. B **47**(1), 558–561 (1993). <https://doi.org/10.1103/physrevb.47.558>
6. G. Kresse, J. Hafner, *Ab initio* molecular-dynamics simulation of the liquid-metal-amorphous-semiconductor transition in germanium. Phys. Rev. B **49**(20), 14251–14269 (1994). <https://doi.org/10.1103/physrevb.49.14251>
7. J. Perdew, K. Burke, M. Ernzerhof, Generalized gradient approximation made simple. Phys. Rev. Lett. **77**(18), 3865–3868 (1996). <https://doi.org/10.1103/PhysRevLett.77.3865>
8. G. Kresse, D. Joubert, From ultrasoft pseudopotentials to the projector augmented-wave method. Phys. Rev. B **59**(3), 1758–1775 (1999). <https://doi.org/10.1103/physrevb.59.1758>
9. S. Grimme, J. Antony, S. Ehrlich, H. Krieg, A consistent and accurate *ab initio* parametrization of density functional dispersion correction (DFT-D) for the 94 elements H-Pu. J. Chem. Phys. **132**(15), 154104 (2010). <https://doi.org/10.1063/1.3382344>
10. K. Momma, F. Izumi, *VESTA 3*for three-dimensional visualization of crystal, volumetric and morphology data. J. Appl. Crystallogr. **44**(6), 1272–1276 (2011). <https://doi.org/10.1107/s0021889811038970>
11. W. Jian, W. Zhang, X. Wei, B. Wu, W. Liang et al., Engineering pore nanostructure of carbon cathodes for zinc ion hybrid supercapacitors. Adv. Funct. Mater. **32**(49), 2209914 (2022). <https://doi.org/10.1002/adfm.202209914>
12. F. Wei, Y. Wei, J. Wang, M. Han, Y. Lv, N, P dual doped foamy-like carbons with abundant defect sites for zinc ion hybrid capacitors. Chem. Eng. J. **450**, 137919 (2022). <https://doi.org/10.1016/j.cej.2022.137919>
13. A. Yu, Y. Zhao, W. Zhang, W. Yang, L. Zhu et al., Bi-directional electrolytic reduction of CO_2_ to mesoporous carbons with regulated structure and surface functional groups for Zn-ion capacitors. Adv. Funct. Mater. **34**(4), 2309666 (2024). <https://doi.org/10.1002/adfm.202309666>
14. Y. Cheng, M. Chen, K. Xia, H. Li, G. Xu et al., Rapid conversion of biomass to hierarchical porous carbons *via* one-step microwave carbonization/activation for long cycle-stable supercapacitor and zinc-ion capacitor. J. Power Sources **624**, 235523 (2024). <https://doi.org/10.1016/j.jpowsour.2024.235523>
15. Z. Tian, C. Yang, C. Zhang, X. Han, J. Han et al., *In-situ* activation of resorcinol-furfural resin derived hierarchical porous carbon for supercapacitors and zinc-ion hybrid capacitors. J. Energy Storage **85**, 111130 (2024). <https://doi.org/10.1016/j.est.2024.111130>
16. Z. Jian, N. Yang, M. Vogel, S. Leith, A. Schulte et al., Flexible diamond fibers for high-energy-density zinc-ion supercapacitors. Adv. Energy Mater. **10**(44), 2002202 (2020). <https://doi.org/10.1002/aenm.202002202>
17. H. Li, P. Su, Q. Liao, Y. Liu, Y. Li et al., Olive leaves-derived hierarchical porous carbon as cathode material for anti-self-discharge zinc-ion hybrid capacitor. Small **19**(49), 2304172 (2023). <https://doi.org/10.1002/smll.20230417>
18. K. Wang, Y. Chen, Y. Liu, H. Zhang, Y. Shen et al., Plasma boosted N, P, O Co-doped carbon microspheres for high performance Zn ion hybrid supercapacitors. J. Alloys Compd. **901**, 163588 (2022). <https://doi.org/10.1016/j.jallcom.2021.163588>
19. H. He, J. Lian, C. Chen, Q. Xiong, C.C. Li et al., Enabling multi-chemisorption sites on carbon nanofibers cathodes by an *in-situ* exfoliation strategy for high-performance Zn-ion hybrid capacitors. Nanomicro Lett. **14**(1), 106 (2022). <https://doi.org/10.1007/s40820-022-00839-z>
